# Probiotics in Irritable Bowel Syndrome: An Umbrella Review of 27 Systematic Reviews on Methodological Quality and Certainty of Evidence

**DOI:** 10.3390/jcm15051727

**Published:** 2026-02-25

**Authors:** Jhosmer Ballena-Caicedo, Fiorella E. Zuzunaga-Montoya, Renzo Acosta-Porzoliz, Félix García-Ahumada, Oriana Rivera-Lozada, Mario J. Valladares-Garrido, Víctor Juan Vera-Ponce

**Affiliations:** 1 Facultad de Medicina (FAMED), Universidad Nacional Toribio Rodríguez de Mendoza de Amazonas (UNTRM), Amazonas, Chachapoyas 01001, Peru; 7330178022@untrm.edu.pe (J.B.-C.); victor.vera@untrm.edu.pe (V.J.V.-P.); 2 Facultad de Medicina, Universidad Continental, Lima 15113, Peru; fiorellazuzunaga@gmail.com; 3 Facultad de Medicina, Universidad Peruana Cayetano Heredia, Lima 15102, Peru; renzo.acosta@upch.pe; 4 EpiHealth Research Center for Epidemiology and Public Health, Lima 15001, Peru; 5 Vicerrectorado de Investigación, Universidad Señor de Sipán, Chiclayo, Lambayeque 14000, Peru; felixgarcia@uss.edu.pe (F.G.-A.); riveraoriana@uss.edu.pe (O.R.-L.)

**Keywords:** probiotics, irritable bowel syndrome, systematic review, meta-analysis, evidence-based medicine

## Abstract

**Background/Objectives**: Irritable bowel syndrome (IBS) is a common and heterogeneous gastrointestinal disorder. Although numerous systematic reviews (SRs) have evaluated the effects of probiotics in IBS, uncertainty persists regarding their clinical effectiveness, methodological quality, and certainty of evidence. This umbrella review aimed to critically appraise SRs on probiotics in IBS, quantify overlap among reviews, and assess the certainty of evidence using the GRADE approach. **Methods**: We conducted an umbrella review of SRs of randomized controlled trials evaluating probiotics in adults with IBS. Searches were performed in MEDLINE/PubMed, Embase, the Cochrane Database of Systematic Reviews, Scopus, and Web of Science from inception to September 2025. Overlap between reviews was assessed using the corrected covered area (CCA). Methodological quality was evaluated with AMSTAR-2, risk of bias with ROBIS, and certainty of evidence with GRADE. **Results**: Twenty-seven SRs published between 2009 and 2025 were included, encompassing 5–82 randomized trials and 243–10,332 participants per review. Methodological quality was low or critically low across all SRs, with 66.7% rated as critically low by AMSTAR-2 and 85.2% judged at high risk of bias by ROBIS. A high degree of overlap was observed between reviews (CCA: 12%). Probiotics were associated with modest improvements in symptom persistence (risk ratio ≈ 0.78–0.79; number needed to treat 4–7), small-to-moderate effects on abdominal pain (standardized mean difference −0.31 to −0.94) with substantial heterogeneity, and small or inconsistent effects on bloating and quality of life. Adverse events were comparable to placebo. Overall, certainty of evidence was predominantly low or very low, with only 1% of outcomes rated as high certainty. **Conclusions**: Although probiotics demonstrate statistically significant benefits for some IBS outcomes, the certainty of evidence remains predominantly low or very low due to methodological limitations, inconsistency, imprecision, and substantial overlap between reviews. The accumulation of redundant SRs has not increased confidence in effect estimates. Future efforts should prioritize well-designed, standardized primary trials rather than additional systematic reviews.

## 1. Introduction

Irritable bowel syndrome (IBS) is a common disorder of gut–brain interaction whose prevalence depends on diagnostic criteria and measurement approach; meta-analyses using historical criteria estimate 11% globally, while more recent syntheses place prevalence at 9.2% with Rome III criteria and 3.8% with Rome IV [1,2]. This condition, defined by recurrent abdominal pain and altered bowel habits, compromises quality of life and generates a substantial economic burden; in the United States, ACG guidelines estimate annual direct costs of approximately USD 1.5–10 billion [3]. Given the clinical heterogeneity and absence of a universally effective treatment, probiotics emerge as a plausible therapeutic option, supported by the gut–brain–microbiota axis hypothesis and evidence of dysbiosis in patients with IBS [4,5].

Research on probiotics in IBS has experienced exponential growth over the past two decades, generating a considerable volume of randomized controlled trials (RCTs). This body of primary evidence has, in turn, motivated the publication of numerous systematic reviews (SRs) seeking to synthesize available findings. However, a preliminary analysis of the literature reveals a concerning phenomenon: the rapid and seemingly uncoordinated expansion of SRs on this same topic. Whereas the early SRs by Brenner et al. [6] and Hoveyda et al. [7] in 2009 addressed an unmet need for evidence synthesis, the subsequent publication of 27 SRs through 2025 suggests possible research redundancy requiring critical evaluation.

A preliminary search of biomedical databases suggests a notable proliferation of SRs on probiotics in IBS in recent years. For instance, a rapid PubMed search using the terms “probiotics,” “irritable bowel syndrome,” and “systematic review” limited to the past five years (2020–2025) yields multiple results, with several reviews published in consecutive years [8,9,10,11,12,13]. This frequent publication pattern raises questions about possible redundancy among these syntheses, particularly when the time elapsed between publications may be insufficient for the generation of significant new primary evidence. Moreover, the existence of multiple systematic reviews on the same topic has been identified as a growing problem in the medical literature, a phenomenon that Ioannidis termed “the mass production of redundant, misleading, and conflicting systematic reviews” [14].

This proliferation of reviews generates a critical problem for evidence-based clinical practice: when multiple systematic syntheses on the same topic yield discordant conclusions or are based on highly overlapping study sets, clinicians face uncertainty about which evidence to use for decision-making. Furthermore, if these reviews present systematic methodological weaknesses, the apparent abundance of evidence may mask limited actual certainty. Given this scenario, the primary objective of this umbrella review (UR) is to evaluate the methodological quality and certainty of evidence of SRs on probiotics for IBS, quantifying overlap among them to determine whether they represent unique contributions or research redundancy. Secondary objectives include the following: (1) synthesizing evidence on the clinical effectiveness and safety of probiotics for key outcomes (global symptoms, abdominal pain, bloating, quality of life); (2) identifying the main causes of certainty downgrading according to GRADE; and (3) establishing recommendations regarding the need for new primary evidence versus additional systematic syntheses.

## 2. Materials and Methods

### 2.1. Design

This UR was conducted following the PRIOR (Preferred Reporting Items for Overviews of Reviews) guidelines for overviews of reviews [15] (see Supplementary Material Table S1), the methodological framework proposed by Aromataris et al. for URs [16] and is reported in alignment with the PRISMA 2020 statement.

### 2.2. Eligibility Criteria

Systematic reviews and meta-analyses evaluating the efficacy of probiotics in the treatment of IBS were included. Reviews were eligible if they: (1) included at least three RCTs; (2) specifically evaluated probiotics (single-strain or multi-strain) versus placebo, standard treatment, or active comparators; (3) included predominantly adult participants (≥18 years) with a diagnosis of IBS (Rome I–IV, Manning, Kruis, or clinical criteria); and (4) reported at least one clinical effectiveness outcome (global symptoms, abdominal pain, bloating, or quality of life). Narrative reviews, scoping reviews, individual patient data meta-analyses, reviews evaluating exclusively prebiotics or synbiotics without separate probiotic analyses, and reviews focused solely on pediatric populations were excluded. No language or publication date restrictions were applied.

### 2.3. Search Strategy

Systematic searches were conducted in MEDLINE (via PubMed), Embase, the Cochrane Database of Systematic Reviews, Scopus, and Web of Science from inception to September 2025. The search strategy combined MeSH terms and free-text words related to “*probiotics*,” “*Lactobacillus*,” “*Bifidobacterium*,” “irritable bowel syndrome,” “IBS,” “systematic review,” and “meta-analysis.” Search strategies were developed with support from a medical librarian and adapted to each database to maximize sensitivity (see Supplementary Material Table S2).

### 2.4. Study Selection and Data Extraction

Three reviewers (JJBC, RAP, and FEZM) independently screened titles and abstracts using the Rayyan platform. Full-text articles were then assessed independently by the same reviewers. Disagreements were resolved by consensus or consultation with a fourth reviewer (VJVP).

Data extraction was performed in duplicate using standardized, piloted forms that captured: review characteristics (author, year, review type); methodological characteristics (e.g., PROSPERO registration, conflict-of-interest reporting); characteristics of included primary studies (number of RCTs, total participants, IBS diagnostic criteria); intervention characteristics (probiotic strains, doses, duration); evaluated outcomes with their effect estimates, 95% confidence intervals, and heterogeneity indices; subgroup or sensitivity analyses; and adverse event assessment.

### 2.5. Data Synthesis and Reporting

Data synthesis followed a structured narrative approach appropriate for umbrella reviews. Systematic reviews and meta-analyses were synthesized according to the clinical outcomes evaluated (global IBS symptoms, abdominal pain, bloating, quality of life, and adverse events) as reported by the included reviews, and when applicable by probiotic characteristics (strain, dose, duration) and IBS subtype. For each outcome, all eligible reviews reporting compatible results were considered for synthesis.

No new quantitative meta-analyses were performed. Effect estimates (risk ratios, mean differences, standardized mean differences), 95% confidence intervals, and heterogeneity measures (I^2^) were extracted as reported by the included reviews. When clearly specified, the primary outcome and time point emphasized by the review authors were extracted. No data transformation or statistical conversion was undertaken; outcomes were synthesized narratively to allow comparison across reviews.

Results of individual reviews and syntheses were tabulated and visually summarized using structured tables provided in the Supplementary Materials. Heterogeneity was explored descriptively based on subgroup analyses reported by the included reviews, including probiotic strain, dose, treatment duration, and IBS subtype. Sensitivity analyses reported by the original reviews were summarized narratively when available.

Risk of bias due to missing results (reporting bias) was not assessed independently at the umbrella review level. Instead, information on publication bias was extracted from the included reviews when reported (e.g., funnel plots or Egger’s tests) and incorporated into the interpretation of findings and the certainty of evidence assessment.

### 2.6. Analysis of Overlap Between Reviews

The degree of overlap among primary studies was assessed using the Corrected Covered Area (CCA) proposed by Pieper et al. [17]. A citation matrix documenting all RCTs included in each systematic review was constructed. CCA was interpreted as 0–5% slight overlap, 6–10% moderate, 11–15% high, and >15% very high. Pairwise overlap indices were additionally calculated to identify direct redundancy between specific reviews.

### 2.7. Assessment of Methodological Quality

Methodological quality was assessed using AMSTAR 2 (A MeaSurement Tool to Assess Systematic Reviews-2) [18], applied independently by three reviewers (JJBC, RAP, and FEZM). Discrepancies were resolved by consensus or consultation with a fourth reviewer (VJVP). AMSTAR 2 consists of 16 items (7 critical and 9 non-critical) and provides an overall confidence rating categorized as high, moderate, low, or critically low. Critical domains include the following: protocol registered a priori, adequacy of the literature search, justification for excluded studies, assessment of risk of bias in included primary studies, appropriateness of meta-analytic methods, consideration of risk of bias when interpreting results, and assessment of publication bias.

### 2.8. Assessment of Risk of Bias in Reviews (ROBIS)

Risk of bias was assessed using the ROBIS tool [19]. Three reviewers (JJBC, RAP, and FEZM) applied ROBIS independently after pilot calibration; disagreements were resolved by consensus or adjudication by a fourth assessor (VJVP). Each item was scored using standardized responses (Yes, Probably yes, Probably no, No, No information), leading to judgments for each domain (low, high, or unclear). The overall ROBIS judgment was classified as low when all domains showed low risk, high when ≥1 critical domain had high risk, and unclear when information was insufficient to judge at least one domain.

### 2.9. Assessment of Certainty of Evidence

Certainty of evidence was assessed using the GRADE approach adapted for umbrella reviews [20]. Because included reviews had already synthesized primary studies, the GRADE assessment focused on the certainty of the body of evidence reported for each outcome, considering the (1) methodological limitations of the review (AMSTAR 2 and ROBIS); (2) consistency across reviews evaluating the same outcome; (3) precision of effect estimates; (4) presence of publication bias when assessed by original reviews; and (5) indirectness related to variations in populations, interventions, or comparators. Certainty was downgraded when limitations in these domains reduced confidence in effect estimates. Independent GRADE assessments of primary studies were not performed. Ratings were conducted independently by three assessors (JJBC, RAP, and FEZM), with discrepancies resolved through discussion or by a fourth assessor (VJVP).

## 3. Results

### 3.1. Study Selection

The systematic search across databases identified 1,768 records: Scopus (628), Embase (527), PubMed (203), Web of Science (405), and Cochrane (5). After removing 447 duplicates, 1321 titles and abstracts were screened, of which 1272 were excluded primarily because they were unrelated to probiotics or IBS, were primary studies, narrative reviews, pediatric-population studies, articles addressing other gastrointestinal conditions, or duplicates not initially detected. Forty-nine full-text articles were assessed for eligibility, and 22 were excluded for the following reasons: narrative reviews without a systematic search (*n* = 3), combined interventions without separate probiotic analyses (n = 12), and exclusive focus on pediatric populations (*n* = 7). Finally, 27 systematic reviews met all eligibility criteria and were included in the qualitative synthesis [6,7,8,9,10,11,12,13,21,22,23,24,25,26,27,28,29,30,31,32,33,34,35,36,37,38,39] (see Supplementary Material Figure S1).

### 3.2. Characteristics of Included Reviews

A total of 27 systematic reviews published between 2009 and 2025 were included. Their annual distribution reflected a progressive increase in publications on the topic (see Supplementary Material Table S3). In 2009, two reviews were published [6,7], followed by one in 2010 [39], one in 2015 [38], one in 2017 [37], two in 2018 [35,36], and two in 2019 [33,34]. From 2020 onward, productivity intensified, with four reviews in 2020 [11,12,13,32], four in 2022 [28,29,30,31], a peak of five in 2023 [10,24,25,26,27], four in 2024 [9,21,22,23], and one in 2025 [8]. Of these reviews, 24 included a meta-analysis [7,8,9,10,11,12,13,21,23,24,25,26,27,28,29,30,31,32,34,35,36,37,38,39], 4 of which were network meta-analyses [21,26,31,34], while 3 were systematic reviews without statistical synthesis [6,22,33]. The volume of primary evidence varied widely, ranging from 5 trials [35,37] to 82 trials [25] and from 243 participants [35] to 10,332 adult participants [25].

Regarding diagnostic criteria, Rome criteria were the most frequently used across reviews, followed by Manning criteria (reported in 10 reviews) [6,7,21,25,26,29,31,32,36,39] and Kruis criteria (4 reviews) [12,29,31,36]. Twenty-three reviews included all IBS subtypes, while two focused exclusively on IBS-D [29,36] and two on IBS-C [11,30]. Only nine reviews reported protocol registration, including five registered in PROSPERO [8,13,21,23,26], one in the Open Science Framework [24], one in protocols.io [27], and two in the Research Registry [11,12]. Six reviews declared industry funding or conflicts of interest [25,26,28,29,36,39].

### 3.3. Interventions and Comparators

Interventions across the included reviews comprised probiotics mainly from the genera Lactobacillus and Bifidobacterium, and to a lesser extent Saccharomyces, Streptococcus, Escherichia coli, and Clostridium. These were administered as both single-strain and multi-strain formulations, including commercially available preparations such as VSL#3, LacClean Gold, Duolac 7s, and Symbioflor. Doses exhibited wide variability, ranging from 10^6^ to 9 × 10^11^ CFU/day, although most trials administered 10^8^ to 10^11^ CFU/day. Treatment duration ranged from 2 to 24 weeks, with most protocols implemented for 4 to 12 weeks. Placebo was the most common comparator across trials. However, some reviews also included studies comparing probiotics with active interventions, such as trimebutine [8], other pharmacologic agents [30], or dietary approaches, including low-FODMAP diets [31]. (See Table 1 and Supplementary Material Table S3).

Section 3.4, Section 3.5, Section 3.6, Section 3.7, Section 3.8 and Section 3.9 present detailed findings for each clinical outcome. For a consolidated summary of effect magnitude and certainty of evidence across outcomes and reviews, readers may refer to Table 2 and Supplementary Material Table S6.

### 3.4. Global Symptom Improvement

#### Overall Effect

Assessment of global symptom improvement was the primary outcome in most reviews, and results consistently favored probiotics (see Supplementary Material Table S4). Several meta-analyses evaluating symptom persistence reported significantly reduced relative risk (RR), with nearly identical point estimates: RR = 0.78 (95%CI 0.71–0.87; I^2^ = 71%; 32 RCTs) in Goodoory et al. [25], RR = 0.79 (95%CI 0.68–0.91; I^2^ = 72%; 21 RCTs) in Ford et al. [36], and RR = 0.79 (95%CI 0.70–0.89; I^2^ = 72%; 23 RCTs) in Niu et al. [12]. When global improvement was analyzed as probability of improvement, results were similarly consistent (RR = 1.52 in 35 RCTs) [32] and (RR = 1.50 in 22 RCTs) [13]. These effect sizes corresponded to numbers needed to treat (NNT) values ranging from 4 [39] to 7 [36].

Analyses based on global symptom scores also indicated small-to-moderate benefits, with standardized mean differences (SMD) of −0.48 in 54 RCTs [21] and −0.55 in 63 RCTs [24]. In contrast, a meta-analysis restricted to six RCTs using exclusively Rome IV criteria found no significant overall effect [27]. Network meta-analyses further compared the relative performance of different formulations. Xie et al. [26] identified Lactobacillus acidophilus DDS-1 and combinations of Bifidobacterium longum with Lactobacillus rhamnosus as most likely effective. Likewise, a network synthesis by Liang et al. [34] reported that a two-strain formulation (DUO) was superior to placebo (RR = 7.46; 95%CI 2.00–32.23).

### 3.5. Subgroup and Sensitivity Analyses

Subgroup analyses evaluated several potential effect modifiers, yielding variable results across reviews. The effect according to formulation type was inconsistent: some reviews reported greater benefit with single-strain probiotics [32], whereas others found superior or more consistent effects with multi-strain formulations [12,29]. No clear dose–response pattern emerged; one review found no differences between high doses (≥1 × 10^10^ CFU/day) and lower doses [32]. Treatment duration also produced heterogeneous findings. One review reported significant benefit only in interventions lasting up to 8 weeks [24], while others observed comparable effects in both short- and long-duration protocols [12,32]. Benefit appeared more consistent in patients with IBS-D [9]. Sensitivity analyses generally supported the robustness of these findings. Significant effects were maintained when restricting analyses to studies with low risk of bias (RR = 0.77; 95%CI 0.67–0.89) [12]. Likewise, exclusion of outlier trials reduced heterogeneity substantially (I^2^ from 79% to 0%) without altering effect direction (SMD = −0.18) [39] (see Supplementary Material Table S4).

### 3.6. Abdominal Pain

#### Overall Effects

Probiotics consistently reduced abdominal pain compared with placebo, although the magnitude and heterogeneity of effects varied notably across reviews. A large synthesis including 48 studies reported a moderate-to-large reduction in pain scores (SMD = −0.89; 95%CI −1.29 to −0.50), albeit with very high heterogeneity (I^2^ = 98.4%) [24]. Other reviews identified only small effect sizes, such as SMD = −0.31 (95%CI −0.44 to −0.17) across 14 RCTs with substantially lower heterogeneity (I^2^ = 24) [36]. When pain was analyzed as persistence rather than continuous score, another review found a significant effect favoring probiotics (RR = 0.72; 95%CI 0.64–0.82; I^2^ = 72%) in 32 RCTs [25]. However, benefits were not uniform across all patient groups. In subpopulations such as IBS-C or in analyses restricted to the strain Bifidobacterium infantis 35624, no significant effect was observed [37]. Additionally, a network meta-analysis encompassing 47 trials identified Bacillus coagulans MTCC 5856 as the formulation most likely to reduce abdominal pain [26].

Subgroup analyses revealed heterogeneous patterns. Probiotics belonging to the genus Bacillus consistently demonstrated the strongest effects (RR = 0.33; SMD = −2.23) [24,25], whereas findings for Lactobacillus and Bifidobacterium were inconsistent across reviews. Multi-strain formulations appeared more effective in IBS-D [28]. Dose-related trends were mixed, although one review suggested greater benefit at ≥10^10^ CFU/day [23]. Treatment duration also influenced results: in some analyses, improvements were more pronounced in interventions lasting ≤8 weeks [24], whereas others found similar effects regardless of duration [12,32]. Sensitivity analyses yielded variable robustness. In one review, restricting analyses to RCTs with low risk of bias preserved a modest but significant effect (SMD = −0.27) [12], whereas in another, the effect became non-significant under the same restriction [27]. The exclusion of outlier studies frequently produced substantial reductions in heterogeneity—for instance, I^2^ decreasing from 85% to 0% while retaining statistical significance [39]. Conversely, in a separate analysis, removal of an influential outlier caused a previously non-significant result to become significant, illustrating the methodological fragility of some pooled estimates [30]

### 3.7. Abdominal Bloating

#### Overall Effect

Results on abdominal bloating were more heterogeneous compared to other outcomes, with several major reviews reporting no significant overall effect of probiotics [21,23,39]. Nevertheless, other meta-analyses documented statistically significant reductions. A review including 26 RCTs found lower bloating persistence (RR = 0.75; 95%CI 0.64–0.88; I^2^ = 78%) [25], while another with 17 studies reported a reduction in symptom score (MD = −2.13; 95%CI −3.96 to −0.30; I^2^ = 99.7%) [9]. Additional reviews identified small but statistically significant effects, with SMD ≈ −0.13 to −0.15 [12,13]. A network meta-analysis of 39 RCTs ranked a combination of Bifidobacterium longum and Lactobacillus rhamnosus as the most likely effective formulation [26].

Subgroup findings were mixed. Probiotics from the genus Bacillus showed a significant effect (RR = 0.41) [25], although this was not consistently replicated with other genera. Benefits were more evident in patients with IBS-D [9], particularly when receiving multi-strain formulations [28], and appeared more consistent in short-duration treatments (<8 weeks) [23,32]. Sensitivity analyses highlighted the fragility of the evidence. In one review, removing a single influential study eliminated the previously significant effect [7]. Risk-of-bias-restricted analyses produced heterogeneous results: in one review, the effect remained significant only in low-risk-of-bias studies [12], whereas another review showed significant effects in both the overall and low-risk analyses [27]. Full details are provided in Supplementary Material Table S4.

### 3.8. Quality of Life

#### Overall Effects

Findings regarding the effect of probiotics on quality of life (QoL) were mixed. Several reviews reported no significant overall improvement [13,28,35]. However, larger meta-analyses demonstrated beneficial effects. One review including 23 RCTs found a large magnitude improvement in QoL (SMD = 0.99; 95%CI 0.45–1.54; I^2^ = 98.0%) [10]. Another meta-analysis of 13 RCTs reported a mean increase of +8.77 points on QoL scales (95%CI +0.91 to +16.64; I^2^ = 99.5%) [9]. A 2024 review found a small but statistically significant improvement (SMD = 0.29; 95%CI 0.15–0.42; I^2^ = 41.9%) [23]. A network meta-analysis identified a specific multi-strain combination and Clostridium butyricum CGMCC0313.1 as the interventions most likely to yield greater QoL improvement [26].

Subgroup analyses revealed notable differences. Saccharomyces-based interventions produced large improvements in some reviews [24], and short-duration treatments (<8 weeks) were also associated with stronger effects. The direction and magnitude of outcomes varied across measurement scales, contributing to heterogeneity. Sensitivity analyses suggested that QoL results were more robust in higher-quality studies. In one review, a previously non-significant overall effect became significant when restricting the analysis to low-risk-of-bias RCTs, yielding SMD = −0.18 (95%CI −0.32 to −0.04; I^2^ = 0%) [27]. Detailed subgroup results are presented in Supplementary Material Table S4.

### 3.9. Adverse Events and Safety

Across the included reviews, probiotics demonstrated a favorable safety profile, with no increased risk of adverse events compared with placebo. Large meta-analyses consistently reported no significant differences in total adverse events: one review including 55 studies reported an RR of 1.05 (95%CI 0.90–1.22; I^2^ = 34%) [25]; another, analyzing 36 randomized trials, reported an RR of 1.09 (95%CI 0.91–1.29; I^2^ = 36%) [36]; and a third review including 40 studies found an RR of 1.07 (95%CI 0.92–1.24; I^2^ = 0%) [32]. An isolated review detected a slightly higher incidence of adverse events in the probiotic group (RR = 1.21; 95%CI 1.02–1.44) [12]. Serious adverse events were rare across all trials, and no review attributed severe events directly to probiotic use [26].

Probiotics were generally well tolerated. Reported adverse events were mild, transient, and predominantly gastrointestinal in nature, including bloating, gas, nausea, or digestive discomfort [6,29]. Several trials explicitly reported the absence of any adverse events; for example, one review described 14 studies in which no events occurred [32]. Overall, the frequency and severity of adverse events were similar between probiotic and placebo groups, supporting the benign safety profile of these interventions.

### 3.10. Overlap Index

To assess redundancy among recent syntheses, we calculated the overlap index for systematic reviews published between 2020 and 2025, the period with the highest concentration of publications (18 reviews). Collectively, these reviews reported 614 citations corresponding to 202 unique randomized trials. Using the study-by-review citation matrix, the CCA was 12.0%, indicating a high degree of overlap.

Pairwise comparisons demonstrated substantial redundancy among several influential reviews. The greatest overlap occurred between Chen 2023 and Xie 2023 [24,26], which shared 57 of the 78 randomized trials included in one review. A similarly high degree of overlap was found between Goodoory 2023 and Xie 2023 [25,26], which shared 57 of 87 studies, and between Chen 2023 and Goodoory 2023 [24,25], which shared 58 of 93 studies. These patterns demonstrate that recent systematic reviews rely on a largely identical set of primary trials. This high overlap indicates that differences in conclusions across reviews are unlikely to be driven by distinct evidence bases. Instead, discrepancies appear to arise from methodological decisions—such as eligibility criteria, outcome definitions, subgroup strategies, and statistical models—rather than from the incorporation of novel primary evidence. Consequently, the proliferation of systematic reviews on probiotics in IBS reflects research redundancy rather than meaningful expansion of the evidence base.

### 3.11. Synthesis of Quality and Certainty of Evidence

**Figure 1:**
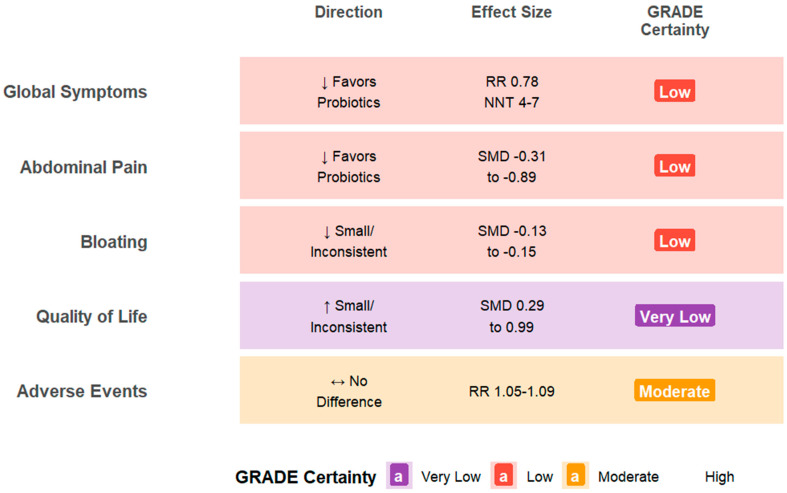
Summary of findings: probiotics vs. placebo in IBS. Visual synthesis of effect direction, magnitude, and GRADE certainty across five clinical outcomes. Row shading reflects predominant GRADE certainty. Arrows indicate effect direction relative to placebo: ↓ favors probiotics (reduction in symptoms), ↑ indicates an increase, and ↔ indicates no difference. Abbreviations: RR, risk ratio; SMD, standardized mean difference; NNT, number needed to treat; SR, systematic review. Data synthesized from 27 systematic reviews (2009–2025). GRADE assessment performed at the umbrella review level.

The GRADE approach was applied to evaluate the certainty of the evidence for 92 outcomes reported across 27 systematic reviews. Overall certainty was limited, with only one outcome (1%) rated as high certainty and 34 (37%) as moderate. By contrast, 57 of 92 outcomes (62%) were classified as low (*n* = 37) or very low (*n* = 20), indicating reduced confidence in most effect estimates (Supplementary Material Table S5). To provide a visual overview of the evidence, Figure 1 synthesizes the direction, magnitude, and GRADE certainty for each clinical outcome across all included reviews. This summary illustrates that while most outcomes favor probiotics over placebo, certainty of evidence is predominantly low or very low. For detailed study-level data, Table 2 presents the 12 reviews with the highest-quality GRADE profiles—those reporting at least two outcomes rated as moderate or higher—while the complete compilation of all 27 reviews is provided in Supplementary Material Table S6.

Analysis by outcome category showed distinct patterns. For global IBS symptoms (28 assessments), certainty was variable, with one high-certainty assessment [34] and 14 rated as moderate, while 13 were downgraded to low or very low, often due to a very serious risk of bias. For abdominal pain (24 assessments), certainty was mainly concentrated at low and very low levels (14 of 24), frequently attributable to inconsistency across primary studies. A similar pattern was observed for abdominal bloating (24 assessments), where only 7 assessments reached moderate certainty. Quality of life (16 assessments) emerged as the outcome with the greatest compromise, as 13 assessments were rated low or very low.

Across outcomes, downgrading was primarily driven by serious concerns regarding inconsistency and risk of bias. Imprecision was the third most frequent cause of downgrade, whereas publication bias contributed to several assessments and indirectness was the least common reason. No upgrading was applied for any outcome (Supplementary Material Table S5).

### 3.12. Assessment of Risk of Bias of the Reviews Using AMSTAR 2

All 27 systematic reviews were evaluated with the AMSTAR 2 tool. Nine reviews (33.3%) were classified as having low methodological confidence, and 18 (66.7%) as critically low, reflecting the presence of weaknesses in at least one critical domain in all reviews, which substantially limits confidence in their conclusions (Supplementary Material Table S7).

Critical domain analysis revealed systematic and widespread deficiencies. Item 7 (list of excluded studies with justification) showed complete non-compliance, with all 27 reviews failing to meet this criterion. Other frequent critical weaknesses were identified in item 15 (consideration of risk of bias when interpreting results; 14 reviews), item 2 (prior protocol; 21 failures), item 4 (comprehensive search; 18 failures), and item 13 (appropriate interpretation of risk of bias; 10 failures). Although performance was comparatively better for item 9 (risk of bias assessment) and item 11 (appropriate statistical methods), deficiencies were still present in 8 and 4 reviews, respectively.

Among non-critical domains, excellent compliance was found for item 1 (research question) and item 16 (conflicts of interest), both met by all reviews, and item 8 (description of included studies), met by 26 reviews. However, significant weaknesses persisted in item 10 (reporting funding sources of included studies), where 25 reviews failed to meet the criterion.

### 3.13. Assessment of Risk of Bias of the Reviews Using ROBIS

The overall ROBIS judgment showed concerning results, with 23 reviews (85.2%) classified as high risk of bias and the remaining 4 (14.8%) flagged with concerns due to unclear information. No review met the criteria for low overall risk, indicating that all included reviews exhibited methodological or reporting issues that may compromise the validity of their conclusions (Supplementary Material Table S8).

Domain-level assessment identified several problem areas. Domain 4 (synthesis and findings) was the most deficient, with 19 reviews rated high risk and none low risk. Domain 2 (study identification and selection) also showed poor performance, with 14 reviews at high risk and 7 at low risk. In contrast, Domain 3 (data collection and study appraisal) demonstrated the best performance, being the only domain with more low-risk (14 of 27) than high-risk ratings (7 of 27). Domain 1 (eligibility criteria) showed a balanced distribution, with 11 reviews rated as high risk and 11 as low risk.

## 4. Discussion

### 4.1. General Interpretation of Findings

This UR reveals a critical paradox in the literature on probiotics for IBS: while the evidence shows statistically significant benefits, the overall certainty supporting these benefits is predominantly low or very low. The GRADE assessment of 92 outcomes demonstrated that only 1% achieved high certainty, 37% moderate, and 62% were of low or very low certainty, mainly due to risk of bias, inconsistency, imprecision, and suspected publication bias. Overlap between reviews was high (CCA 12% for 2020–2025), indicating that multiple syntheses relied on a largely shared pool of RCTs and therefore added little incremental knowledge. Regarding effect magnitude, probiotics demonstrated modest benefits in global symptoms (equivalent to NNT 4–7) and small-to-moderate effects on abdominal pain. Effects on bloating were generally small or non-significant, and quality of life outcomes showed inconsistent and heterogeneous results across reviews. Network meta-analyses identified differential signals by strain (Bacillus coagulans MTCC 5856 for pain; Bifidobacterium longum + Lactobacillus rhamnosus for bloating), although these should be considered hypothesis-generating given the high heterogeneity and absence of confirmatory RCTs. The safety profile was favorable, with no significant differences in adverse events versus placebo.

The apparent abundance of evidence on probiotics in IBS masks limited certainty for clinical decision-making. The proliferation of redundant systematic reviews has not increased confidence in effect estimates; instead, it has generated artificial variability in conclusions without resolving fundamental uncertainties about effect magnitude, responder populations, and optimal strain selection. This phenomenon of “meta-production without knowledge gain” has important implications: the path forward does not require more synthesis of existing evidence, but rather new high-quality primary evidence (RCTs with standardized outcomes, stratification by IBS phenotype, integration of biomarkers) that elevates certainty and enables more definitive clinical recommendations. These findings suggest that the effect of probiotics exists but is fragile and highly dependent on analytical decisions. Uncertainty remains about its real magnitude and clinical applicability, justifying a prudent stance that avoids both therapeutic nihilism and inflated expectations.

### 4.2. Biological Interpretation (Microbiota–Gut–Brain Axis)

From a pathophysiological perspective, the observed effects are biologically plausible within the microbiota–gut–brain axis framework. Lactobacillus and Bifidobacterium can modulate key metabolites such as short-chain fatty acids (SCFAs), bile acid biotransformation via hydrolases (BSH), and tryptophan/indole pathways, with impacts on motility, visceral sensitivity, and permeability [40,41]. The microbiota (especially sporulated bacteria) regulates serotonin biosynthesis by enterochromaffin cells, supporting a plausible link with pain and bowel habits in IBS; this 5-HT-dependent axis has been demonstrated experimentally and provides mechanistic coherence to the modest clinical changes described in this UR [42].

Epithelial barriers and permeability constitute another common pathophysiological substrate. Various probiotic strains have demonstrated preservation of tight junctions (occludin/claudins), reduction of leakage markers such as zonulin, and restoration of epithelial integrity in preclinical models and organ-on-chip systems, which could translate into reduced bacterial translocation and attenuation of peripheral nociceptive stimuli. The recent literature reinforces the role of tight junction proteins and mucins (MUC2) in mucus layer homeostasis; furthermore, butyrate—a product of microbial cross-feeding—can modulate MUC expression and favor goblet cell renewal, integrating a mechanism that links microbiota with bloating, visceral sensitivity, and stool consistency [43,44].

At the immunological level, Lactobacillus, Bifidobacterium, and Saccharomyces can attenuate TLR/NF-κB pathways and shift the cytokine profile toward a more regulatory and anti-inflammatory state (↑IL-10; ↓IL-6/TNF-α), with participation of mast cells in the lamina propria—cells implicated in visceral hypersensitivity, a cardinal phenomenon of IBS [45,46]. In an RCT in IBS-D, Saccharomyces boulardii reduced IL-8/TNF-α and increased IL-10, supporting the translational plausibility of effects on pain and bloating [47].

Finally, vagal signaling and enteric reflexes represent bidirectional communication routes within this axis. In murine models, L. rhamnosus JB-1 modulated central GABA receptors and reduced corticosterone via the vagus nerve, framing a potential mechanism for the modest and variable improvement of pain and quality of life observed in people with IBS. Although extrapolating species/strains and doses from animal models requires caution, this biological evidence adds plausibility to the modest clinical effects reported [48].

Despite this mechanistic plausibility, several factors may explain the modest translation of preclinical findings into clinical efficacy. First, animal models of visceral hypersensitivity or dysbiosis do not fully replicate the heterogeneity of human IBS, which encompasses distinct subtypes, variable symptom severity, and diverse baseline microbiota compositions. Second, probiotic formulations used in RCTs vary considerably in strain selection, dosage, viability, and delivery vehicle, whereas mechanistic studies typically employ standardized conditions. Third, IBS trials consistently report placebo response rates of 27–40% depending on the endpoint used [49], which may attenuate detectable treatment effects. Fourth, clinical outcomes in IBS rely predominantly on subjective symptom scales rather than objective biomarkers, introducing measurement variability that mechanistic studies avoid. Finally, current trials do not stratify patients by microbiome profile or predictive biomarkers, potentially diluting effects that might be substantial in specific responder subpopulations. These translational gaps underscore the need for precision approaches that match specific probiotic strains to defined patient phenotypes.

### 4.3. Comparison with Previous Literature and Clinical Guidelines

These findings are partially concordant with guidelines and consensus documents. The AGA guideline (2020) [50] on probiotics does not recommend their routine use for IBS, given the insufficiency/heterogeneity of evidence and the strain–indication specificity required for clinical benefit, emphasizing the need to link specific strains with demonstrated effects. The ACG guideline for IBS [3] suggests not using probiotics for global symptom relief (conditional recommendation, very low-quality evidence). In contrast, the BSG (2021) [51] admits the possibility of a short therapeutic trial with probiotics, with discontinuation if there is no response. The WGO [50] recognizes a pattern of modest reductions in bloating/flatulence and potential strain-specific benefits, emphasizing the need to select preparations with human evidence. Collectively, guidelines converge on a cautious and conditional positioning, with recommendations that depend on strain/formulation-specific evidence and individual response, aligning with the pattern of modest benefits and limited certainty observed in this UR.

Regarding the recent literature, this UR did not identify substantive changes in the overall interpretation: the most recent reviews, despite increasing study volume, did not significantly modify effect size or elevate GRADE certainty, instead reproducing the heterogeneity and methodological concerns already noted in earlier syntheses. This suggests a marginal and incremental information gain in a high-redundancy environment.

### 4.4. Methodological Analysis and Certainty of Evidence

Formal assessment confirms that certainty of evidence is the main bottleneck for clinical applicability. In AMSTAR 2, the overall judgment was predominantly “critically low” or “low,” with no reviews achieving high or moderate confidence. The most fragile critical domains were as follows: absence of a priori protocol, suboptimal search, lack of a list of excluded studies, insufficient assessment/use of RCT risk of bias when interpreting results, inappropriate meta-analytical methods, and incomplete assessment of publication bias. In ROBIS, no review was classified as low overall risk; 85.2% were considered high risk, with the greatest impairment in the domains of internal validity and presentation of results, as well as in the identification/selection of studies. These methodological deficiencies lead to GRADE downgrading due to risk of bias and publication bias.

Inconsistency between reviews, indicated by high I^2^ and discordant results between instruments/definitions, along with imprecision (wide CIs, modest sample sizes, and susceptibility to sensitivity analyses), prompted additional GRADE downgrades. Indirectness emerged in heterogeneous comparators, scale variability, and absence of standardization by phenotypes (IBS-D/IBS-C/IBS-M). In sum, the GRADE distribution (1% high; 37% moderate; 62% low/very low) highlights that, even when the pooled effect favors probiotics, confidence in that estimate is substantially constrained for most outcomes.

The overlap quantified by CCA illustrates a pattern of “meta-proliferation” without corresponding gains in certainty: multiple reviews essentially recycle the same RCT base, with divergent analytical decisions that generate variability rather than new information. As Ioannidis warned [14], the proliferation of redundant reviews can inflate the perception of consensus without elevating evidence quality, a phenomenon empirically supported in this UR. This finding has implications for evidence-based medicine: iterative synthesis, if not accompanied by methodological improvements and new robust primary evidence, is unlikely to increase certainty.

### 4.5. Clinical and Research Implications

In light of this UR’s results, the clinical message must be prudent: the apparent efficacy of probiotics in IBS is not supported by sufficient evidence certainty. Effect magnitude is, at best, modest and highly dependent on analytical decisions. Certainty is predominantly low or very low due to the risk of bias, inconsistency, imprecision, and suspected publication bias, so a routine recommendation for global symptom relief in general clinical practice is not justified. Consistent with guidelines, use could be considered, at most, as a brief and de-escalable therapeutic trial, with explicit patient information about benefit uncertainty and absence of equivalence between strains/formulations, avoiding extrapolations between products. Signals for specific combinations (e.g., *B. coagulans* MTCC 5856 for pain; *B. longum* + *L. rhamnosus* for bloating) should be interpreted as hypothesis-generating and not as efficacy conclusions translatable to practice, pending confirmation in well-designed and reproducible RCTs.

From a research perspective, the findings support prioritizing new primary evidence over the production of more reviews. Specifically: randomized RCTs with rigorous concealment and blinding; clinically relevant comparators; standardization of diagnostic criteria and outcomes (dichotomous/continuous with clinically interpretable thresholds); and pre-registered protocols with prespecified subgroup analyses by phenotype (IBS-D/IBS-C/IBS-M) and by strain/dose/duration. Additionally, and consistent with the phenomenon of redundant proliferation of systematic reviews, it is reasonable to propose a temporary methodological moratorium on new SRs/MAs regarding probiotics in IBS until robust RCTs emerge that can elevate certainty and change interpretation.

### 4.6. Strengths and Limitations

This UR presents significant methodological strengths that reinforce the validity of its findings. We employed a robust systematic protocol adherent to PRIOR guidelines, with a comprehensive search across five databases without language restrictions, and implemented rigorous methodological assessments using three validated tools (AMSTAR 2, ROBIS, and GRADE). We explicitly quantified overlap between reviews using corrected covered area, empirically demonstrating redundancy in the literature. Finally, this work critically addresses the phenomenon of redundant review proliferation, demonstrating that accelerated review production does not lead to improvements in certainty, with important implications for editors, funders, and researchers regarding the need to redirect resources toward high-quality primary research.

This UR also presents limitations inherent to secondary synthesis. Heterogeneity between reviews (eligibility criteria, outcome definitions, scales, and analytical strategies) hampered the synthesis and direct comparison of effect estimates. GRADE ratings were applied at the systematic review level and not directly to individual primary studies, introducing an additional layer of interpretation that partially depends on the prior judgments of original authors. CCA quantifies overlap and reduces the risk of double-counting, but does not eliminate it nor capture differences in questions or approaches between similar reviews. Individual patient data meta-analysis or trial-level re-extraction was not performed, so it was not possible to explore effect modifiers (age, sex, IBS subtype) or differentiate effects by strain or formulation with precision. Independent assessments of publication bias across primary studies were also not conducted. Finally, although we identified substantial methodological deficiencies, we cannot definitively determine whether these deficiencies altered conclusions about effectiveness, although the very low certainty of evidence suggests caution in interpretation.

## 5. Conclusions and Recommendations

Probiotics confer modest clinical benefits in irritable bowel syndrome, with small but measurable reductions in global symptoms, pain, and abdominal bloating, and a safety profile comparable to placebo. However, the certainty of this evidence is predominantly low or very low due to systematic methodological weaknesses in existing reviews, high inconsistency between studies, and substantial overlap reflecting research redundancy without net knowledge gain. The apparent abundance of syntheses on probiotics in IBS masks fundamental uncertainty about the real magnitude of effect, responder populations, and optimal selection of strains and doses. Consequently, routine recommendation in clinical practice is not justified; at most, a brief therapeutic trial (4–8 weeks) could be considered in selected patients who have failed conventional treatments, using products with specific evidence in clinical trials and with transparent patient information about benefit uncertainty. Specific strain-level recommendations cannot be provided because the signals identified for individual probiotics (e.g., *Bacillus coagulans* MTCC 5856 for pain, *Bifidobacterium longum* combined with *Lactobacillus rhamnosus* for bloating) derive from network meta-analyses with high heterogeneity and have not been confirmed in independent, adequately powered trials.

For clinicians, routine prescription of probiotics as first-line treatment for IBS should be avoided. Consider their use as an individualized therapeutic trial in refractory patients, with discontinuation if there is no subjective response after 8 weeks, and without assuming equivalence between commercial products. For researchers, the findings underscore the need to prioritize new robust primary evidence over the production of additional systematic reviews. Pragmatic clinical trials with standardized outcomes, stratification by IBS phenotype (diarrhea/constipation/mixed), integration of predictive response biomarkers, and clinically relevant comparators (not only placebo) are required. Given the identified redundancy, we propose a temporary methodological moratorium on new systematic reviews until high-quality trials emerge that modify current certainty. Finally, for editors and funders, it is necessary to implement policies that discourage the publication of redundant systematic reviews by requiring explicit justification for the need for new syntheses, prospective quantification of expected overlap, and demonstration of questions not answered by previous syntheses. Redirect editorial and funding resources toward primary research that elevates certainty of evidence and enables definitive clinical recommendations.

## Figures and Tables

**Table 1:** Characteristics of probiotic interventions, doses, duration, and comparators used in included systematic reviews.

First Author, Year	Main Probiotic Strains	Formulation (Single/Multi-Strain)	Dose (CFU/Day)	Duration (Weeks)	Primary Comparator
Yu Q-X, 2025 [8]	*Bifidobacterium, Lactobacillus, Bacillus, Saccharomyces*	Single-strain and combinations	Probiotics: 0.5–1.5 g BID-TID	4–8	Trimebutine alone (100–200 mg TID)
Almabruk BA, 2024 [9]	*Lactobacillus, Bifidobacterium, E. coli, S. boulardii, S. cerevisiae*	Single- and multi-strain	10^7^–10^11^	2–24 (majority 12–16 for multi-strain)	Placebo
Wu Y, 2024 [21]	*Bifidobacterium, Lactobacillus, Saccharomyces*	Single- and multi-strain	10^8^–10^11^ (majority)	4–24 (majority 4–12)	Placebo
Umeano L, 2024 [22]	*Bacillus, Lactobacillus, Bifidobacterium*	Single- and multi-strain	10^9^–10^10^	4–16	Placebo
Yang R, 2024 [23]	*S. boulardii, B. infantis, L. plantarum*	Single-strain and combinations	10^8^–10^11^	4–24	Placebo
Chen M, 2023 [24]	*Lactobacillus, Bifidobacterium, Bacillus, Enterococcus, E. coli, Saccharomyces*	Single-strain and combinations	10^7^–10^11^	4–8 (majority)	Placebo
Goodoory VC, 2023 [25]	*Lactobacillus, Saccharomyces, Bifidobacterium, Bacillus, E. coli, Streptococcus, Blautia, Clostridium*	Single-strain and combinations	10^7^–10^11^	≥7 days treatment and follow-up (majority 2–8)	Placebo
Qing Q, 2023 [10]	*Saccharomyces (S. boulardii, S. cerevisiae)*	Single-strain	250 mg BID–1000 mg QD (10^9^–10^11^)	4–12	Placebo
Xie P, 2023 [26]	*Lactobacillus, Bifidobacterium, Bacillus, Saccharomyces, Clostridium*	Single- and multi-strain	10^9^–10^11^ (usually)	2–24 (majority 4–12)	Placebo
Konstantis G, 2023 [27]	9 types: *B. coagulans, B. longum, L. rhamnosus, L. acidophilus, B. lactis, S. thermophilus, L. plantarum, B. breve, S. cerevisiae*	Single-strain	10^9^–10^10^	3–8.5	Placebo
Wang Y, 2022 [28]	*Lactobacillus, Bifidobacterium, Streptococcus, Saccharomyces, Clostridium, Bacillus*; VSL#3	Single- and multi-strain	8 × 10^8^–9 × 10^11^	4–16	Placebo
van der Geest AM, 2022 [29]	*Bifidobacterium, Saccharomyces, Lactobacillus, Clostridium*	Single- and multi-strain	10^6^–10^11^	4–16	Active drugs
Shang X, 2022 [30]	*Lactobacillus, Bifidobacterium, Saccharomyces, Streptococcus*	Single-strain	10^8^–10^10^	4–12	Placebo
Xie CR, 2022 [31]	*Lactobacillus, Bifidobacterium, Bacillus, Saccharomyces, E. coli, Enterococcus*	Single- and multi-strain	Variable by strain	2–48 (majority 4–8)	Placebo or low-FODMAP diet
Wen Y, 2020 [11]	*B. lactis, Lactobacillus, Streptococcus, E. coli*	Single-strain	10^8^–10^11^	2–12 (majority 2–4)	Placebo
Li B, 2020 [32]	*Lactobacillus, Bifidobacterium, Saccharomyces, Clostridium, Streptococcus, Enterococcus, E. coli*	Single- and multi-strain	10^7^–10^11^	3–24	Placebo
Niu HL, 2020 [12]	*Lactobacillus, Bifidobacterium, E. coli, S. boulardii, Streptococcus*	Single- and multi-strain	10^7^–10^11^	4–20 (majority 4)	Placebo
Sun JR, 2020 [13]	*Lactobacillus, Bifidobacterium, E. coli, S. cerevisiae*	Single-strain and combinations	10^6^–10^11^	4–24 (majority 4–12)	Placebo
Dale HF, 2019 [33]	*L. acidophilus, B. coagulans* MTCC5856, *S. cerevisiae* CNCM I-3856; commercial multi-strain	Single- and multi-strain	10^9^ to >10^11^	4–16	Placebo
Liang D, 2019 [34]	Combinations (DUO, LAC, PRO, F19, Bif)	Single-strain and combinations	0.0001 × 10^10^–7.5 × 10^10^	4–12	Placebo
Connell M, 2018 [35]	VSL#3 (*Bifidobacterium*, *Lactobacillus*, *Streptococcus*)	Multi-strain	450–900 × 10^9^	4–8	Placebo
Ford AC, 2018 [36]	*Lactobacillus, Saccharomyces, E. coli, Streptococcus*	Single-strain and combinations	10^8^–10^11^	4–24 (some up to 6 months)	Placebo
Yuan F, 2017 [37]	*B. infantis* 35624 (single-strain or in combination)	Single-strain and combination	10^8^–10^10^	4–8	Placebo
Didari T, 2015 [38]	*Bifidobacterium*, *Lactobacillus*, *E. coli*; VSL#3, Symbioflor	Single-strain and combinations	10^7^–10^11^	3–20 with follow-up	Placebo
Moayyedi P, 2010 [39]	*Lactobacillus, Bifidobacterium, Streptococcus*; VSL#3	Single- and multi-strain	10^6^–10^10^	2–24	Placebo
Brenner DM, 2009 [6]	*Bifidobacterium, Lactobacillus*; VSL, Prescript Assist, SCM III	Single- and multi-strain	10^6^–10^10^	4–24	Placebo
Hoveyda N, 2009 [7]	*Bifidobacterium, Lactobacillus*; VSL#3	Single- and multi-strain	10^6^–10^10^ single-strain; up to 900 × 10^9^ VSL#3	4–24 (majority 4–8)	Placebo

**Table 2:** Clinical efficacy of probiotics in irritable bowel syndrome according to primary outcomes.

Study (Year)	General Symptoms	Abdominal Pain	Abdominal Bloating	Quality of Life
Wu (2024) [21]	--- ---	↑ ⊕⊖⊖⊖	↔ ⊕⊕⊖⊖	--- ---
Yang (2024) [23]	↑↑ ⊕⊕⊖⊖	↑ ⊕⊕⊖⊖	↔ ⊕⊕⊖⊖	--- ---
Qing (2023) [10]	--- ---	↑ ⊕⊕⊖⊖	↔ ⊕⊕⊖⊖	↑ ⊕⊕⊖⊖
Xie (2023) [26]	--- ---	↑↑ ⊕⊕⊖⊖	--- ---	--- ---
Van der Geest (2022) [29]	↑ ⊕⊕⊖⊖	--- ---	↔ ⊕⊕⊖⊖	--- ---
Li (2020) [32]	--- ---	--- ---	↑ ⊕⊕⊖⊖	--- ---
Sun (2020) [13]	--- ---	↑ ⊕⊕⊖⊖	↑ ⊕⊕⊖⊖	↑ ⊕⊕⊖⊖
Dale (2019) [33]	↑↑ ⊕⊕⊖⊖	↑ ⊕⊕⊖⊖	↑ ⊕⊕⊖⊖	--- ---
Ford (2018) [36]	↑ ⊕⊕⊕⊖	--- ---	↑ ⊕⊕⊖⊖	--- ---
Moayyedi (2010) [39]	↑ ⊕⊕⊖⊖	--- ---	↑ ⊕⊕⊖⊖	--- ---
Brenner (2009) [6]	↑↑ ⊕⊕⊕⊖	--- ---	--- ---	--- ---
Hoveyda (2009) [7]	↑↑ ⊕⊕⊖⊖	--- ---	↑↑ ⊕⊕⊖⊖	--- ---

Legend: Synthesis of probiotic efficacy for four clinical outcomes based on 12 systematic reviews with at least two outcomes rated moderate or higher by GRADE. Effect magnitude: ↑↑↑ large (OR/RR > 2.0 or <0.5; |SMD| > 0.8); ↑↑ moderate (OR/RR 1.5–2.0 or 0.5–0.67; |SMD| 0.5–0.8); ↑ small (OR/RR 1.2–1.5 or 0.67–0.83; |SMD| 0.2–0.5); ↔ no significant effect; --- not reported. GRADE certainty: ⊕⊕⊕⊕ high; ⊕⊕⊕⊖ moderate; ⊕⊕⊖⊖ low; ⊕⊖⊖⊖ very low. Complete data for all 27 reviews available in Supplementary Table S6. Abbreviations: OR, odds ratio; RR, risk ratio; SMD, standardized mean difference; GRADE, Grading of Recommendations Assessment, Development and Evaluation.

## Data Availability

All data analyzed in this umbrella review are included in the published article and its Supplementary Materials. The dataset consists exclusively of data extracted from previously published systematic reviews, which are publicly available and appropriately cited in the manuscript. No additional unpublished data were generated.

## Supplementary Materials

The following supporting information can be downloaded at: https://www.mdpi.com/article/10.3390/jcm15051727/s1, Table S1: PRIOR statement—reporting guideline for overviews of reviews; Table S2: Complete electronic search strategy for all databases; Figure S1: Flowchart of study selection; Table S3: Methodological characteristics of included systematic reviews; Table S4: Main findings of each included systematic review, sensitivity analyses, and reported adverse events; Table S5: Downgrading the certainty of evidence according to GRADE domains (risk of bias, inconsistency, indirectness, imprecision, and publication bias) for the 92 outcomes evaluated across the 27 systematic reviews; Table S6: Complete evaluation of clinical efficacy and methodological quality of the 27 included systematic reviews; Table S7: AMSTAR-2 methodological quality assessment of included systematic reviews; Table S8: ROBIS risk of bias assessment of included systematic reviews; Table S9: PRISMA 2020 checklist. Ref. [52] is listed in the supplementary.

## Abbreviations

The following abbreviations are used in this manuscript:

ACG	American College of Gastroenterology
AGA	American Gastroenterological Association
AMSTAR 2	A MeaSurement Tool to Assess Systematic Reviews 2
APC	Article processing charge
BSG	British Society of Gastroenterology
BSH	Bile salt hydrolase
CCA	corrected covered area
CFU	colony-forming units
CI	confidence interval
CONSORT	Consolidated Standards of Reporting Trials
COVID-19	coronavirus disease 2019
IBS	irritable bowel syndrome
IBS-C	constipation-predominant irritable bowel syndrome
IBS-D	diarrhea-predominant irritable bowel syndrome
IBS-M	mixed-type irritable bowel syndrome
ICHPPC/WONCA	International Classification of Health Problems in Primary Care/World Organization of National Colleges, Academies and Academic Associations of General Practitioners/Family Physicians
IRB	Institutional Review Board
MA	meta-analysis
MD	mean difference
MeSH	Medical Subject Headings
NMA	network meta-analysis
NNT	number needed to treat
OR	odds ratio
OSF	Open Science Framework
PRISMA	Preferred Reporting Items for Systematic Reviews and Meta-Analyses
PRIOR	Preferred Reporting Items for Overviews of Reviews
PROSPERO	International Prospective Register of Systematic Reviews
QoL	quality of life
RCT	randomized controlled trial
RR	risk ratio
ROBIS	Risk Of Bias In Systematic reviews
SCFA	short-chain fatty acids
SMD	standardized mean difference
SR	systematic review
TLR	Toll-like receptor
TNF-α	tumor necrosis factor alpha
UR	umbrella review
WGO	World Gastroenterology Organization
