# Supplementary material for: Probiotics in Irritable Bowel Syndrome: An Umbrella Review of 27 Systematic Reviews on Methodological Quality and Certainty of Evidence
Source: J Clin Med. 2026 Feb 25;15(5):1727. doi: 10.3390/jcm15051727 (PMC12985868; doi:10.3390/jcm15051727)
Supplement: Supplementary file 1 [file jcm-15-01727-s001.zip › Supplementary Material/Table S1.docx]

**Supplementary material Table 1.** PRIOR statement—a reporting guideline for overviews of reviews

| **Section** | **Item No.** | **PRIOR Item** | **Compliant** | **Location (page/section)** |
| --- | --- | --- | --- | --- |
| TITLE | 1 | Identify the report as an overview of reviews. | Yes | Probiotics in Irritable Bowel Syndrome: An Umbrella Review of 27 Systematic Reviews on Methodological Quality and Certainty of Evidence |
| ABSTRACT | 2 | Provide a comprehensive summary of objectives, methods, and main results. | Yes | Abstract |
| INTRODUCTION | 3 | Describe the rationale in the context of existing knowledge. | Yes | Introduction – first paragraphs |
| INTRODUCTION | 4 | State the objectives or research questions explicitly. | Yes | End of Introduction – four objectives clearly listed |
| METHODS | 5a | Specify inclusion and exclusion criteria. | Yes | Eligibility Criteria section |
| METHODS | 5b | Define 'systematic review' as used in the inclusion criteria. | Partial | Implicitly defined: systematic reviews and meta-analyses with predefined methods |
| METHODS | 6 | Specify all databases, registers, and date last searched. | Yes | Search Strategy – PubMed, Embase, Cochrane, Web of Science, Epistemonikos (until Jan 2025) |
| METHODS | 7 | Present full search strategies for all databases. | Yes | Supplementary Material 2 – detailed search strategies |
| METHODS | 8a | Describe the methods used to select eligible reviews. | Yes | Study Selection and Data Extraction section – Rayyan screening, independent reviewers |
| METHODS | 8b | Describe how overlap between systematic reviews was identified and managed. | Yes | Overlap Analysis – Corrected Covered Area (CCA) calculated |
| METHODS | 9a | Describe data collection methods. | Yes | Study Selection and Data Extraction – standardized forms and dual extraction |
| METHODS | 9b | Describe methods used to identify and manage primary study overlap at outcome level. | Yes | Overlap Analysis – cross-citation matrix and CCA interpretation |
| METHODS | 9c | Specify methods used to manage discrepant data across reviews. | Yes | Consensus or third-reviewer arbitration |
| METHODS | 10 | List and define all data items and outcomes extracted; note assumptions. | Yes | Data Extraction section – variables, outcomes, assumptions |
| METHODS | 11a | Describe methods used to assess risk of bias or methodological quality of reviews. | Yes | AMSTAR-2 evaluation section |
| METHODS | 11b | Describe how risk of bias of primary studies was collected from reviews. | Yes | AMSTAR-2 item 9, extraction of tools used (Cochrane, Jadad) |
| METHODS | 11c | Describe methods used to assess RoB of supplemental primary studies (if included). | N/A | Not applicable – no supplemental primary studies |
| METHODS | 12a | Describe synthesis methods and rationale. | Yes | Data Synthesis and Analysis section – structured narrative synthesis |
| METHODS | 12b | Describe methods used to explore heterogeneity. | Yes | Heterogeneity by probiotic strain, dose, and IBS subtype described |
| METHODS | 12c | Describe any sensitivity analyses conducted. | Yes | Subgroup and sensitivity analyses per strain/dose |
| METHODS | 13 | Describe assessment of reporting bias. | Yes | AMSTAR-2 item 15; publication bias (Egger, funnel) reported |
| METHODS | 14 | Describe methods to assess certainty/confidence (GRADE). | Yes | Certainty of evidence (GRADE adapted for umbrella reviews) |
| RESULTS | 15a | Describe results of search and selection process; include flow diagram. | Yes | Results – Study selection + Figure 1 (flowchart) |
| RESULTS | 15b | Provide list of excluded studies with reasons. | Yes | Results – Excluded 20 full-texts with reasons |
| RESULTS | 16 | Cite each included review and present characteristics. | Yes | Table 1 – Systematic reviews and meta-analyses (2009–2025) |
| RESULTS | 17 | Describe extent of primary study overlap. | Yes | Overlap Analysis section – CCA = 23.7% |
| RESULTS | 18a | Present quality/risk of bias assessment for each included review. | Yes | Supplementary Material 9 – AMSTAR-2 table |
| RESULTS | 18b | Present risk of bias of primary studies (collected from reviews). | Yes | Reported under AMSTAR-2 item 9 summary |
| RESULTS | 18c | Present risk of bias of supplemental primary studies (if included). | N/A | Not aplicable |
| RESULTS | 19a | Summarize evidence for all outcomes, with effect estimates and heterogeneity. | Yes | Results – Effectiveness of probiotics; Table 2 (GRADE) |
| RESULTS | 19b | Report investigations of heterogeneity causes. | Yes | Heterogeneity in criteria and populations section |
| RESULTS | 19c | Report sensitivity analyses. | Yes | Subgroup and sensitivity analysis (strain/dose/duration) |
| RESULTS | 20 | Present assessments of reporting bias for each synthesis. | Yes | AMSTAR-2 (item 15) summarized |
| RESULTS | 21 | Present certainty/confidence in evidence for each outcome. | Yes | Table 3 – GRADE evaluation |
| DISCUSSION | 22a | Summarize main findings and discrepancies. | Yes | Discussion – Main findings and interpretation |
| DISCUSSION | 22b | Interpret results in context of other evidence. | Yes | Discussion – Saturation phenomenon and literature comparison |
| DISCUSSION | 22c | Discuss limitations of the evidence and overview methods. | Yes | Limitations section |
| DISCUSSION | 22d | Discuss implications for practice, policy, and future research. | Yes | Implications for practice and future research |
| OTHER INFO | 23a | Provide registration information or state unregistered. | No | Unregistered – stated in limitations |
| OTHER INFO | 23b | Indicate where protocol can be accessed or note absence. | Yes | Methods – Followed PRIOR and Aromataris framework; no PROSPERO registration |
| OTHER INFO | 23c | Describe amendments to registration/protocol. | N/A | Not aplicable |
| OTHER INFO | 24 | Describe funding sources and sponsor roles. | Yes | Funding – Vicerrectorado de Investigación UNTRM |
| OTHER INFO | 25 | Declare competing interests. | Yes | Conflict of interest – none declared |
| OTHER INFO | 26a | Provide corresponding author contact. | Yes | Title page (emails + ORCIDs) |
| OTHER INFO | 26b | Describe author contributions and guarantor. | Yes | Authors’ contribution section |
| OTHER INFO | 27 | Report data, forms, code availability, and access conditions. | Partial | Data available on request – no public repository |
