# Supplementary material for: Probiotics in Irritable Bowel Syndrome: An Umbrella Review of 27 Systematic Reviews on Methodological Quality and Certainty of Evidence
Source: J Clin Med. 2026 Feb 25;15(5):1727. doi: 10.3390/jcm15051727 (PMC12985868; doi:10.3390/jcm15051727)
Supplement: Supplementary file 1 [file jcm-15-01727-s001.zip › Supplementary Material/Table S2.docx]

**Supplementary material Table 2.** Search strategy

| Search strategy in PUBMED | |
| --- | --- |
| #1 | "probiotics"[MeSH] OR "probiotic"[tiab] OR "probiotics"[tiab] OR "lactobacillus"[MeSH] OR "lactobacillus"[tiab] OR "bifidobacterium"[MeSH] OR "bifidobacterium"[tiab] OR "saccharomyces"[tiab] OR "bacillus"[tiab] OR "streptococcus thermophilus"[tiab] OR "enterococcus"[tiab] OR "lactococcus"[tiab] OR "probiotic bacteria"[tiab] OR "probiotic agent"[tiab] OR "probiotic agents"[tiab] |
| #2 | "irritable bowel syndrome"[MeSH] OR "irritable bowel syndrome"[tiab] OR "IBS"[tiab] OR "spastic colon"[tiab] OR "irritable colon"[tiab] OR "functional bowel disorder"[tiab] OR "mucous colitis"[tiab] OR "irritable bowel"[tiab] OR "spastic colitis"[tiab] OR "irritable bowel disease"[tiab] OR "IBS-D"[tiab] OR "IBS-C"[tiab] OR "IBS-M"[tiab] OR "diarrhea predominant"[tiab] OR "constipation predominant"[tiab] OR "mixed type IBS"[tiab] |
| #3 | "systematic review"[tiab] OR "systematic reviews"[tiab] OR "meta-analysis"[tiab] OR "meta-analyses"[tiab] OR "meta analysis"[tiab] OR "metaanalysis"[tiab] OR "revisión sistemática"[tiab] OR "revisiones sistemáticas"[tiab] OR "metaanálisis"[tiab] OR "meta análisis"[tiab] OR "overview of reviews"[tiab] OR "umbrella review"[tiab] OR "umbrella reviews"[tiab] OR "review of reviews"[tiab] OR "scoping review"[tiab] OR "narrative review"[tiab] OR "pooled analysis"[tiab] |
| #4 | #1 AND #2 AND #3 |
| Search strategy in SCOPUS | |
| #1 | TITLE-ABS-KEY ("probiotic*" OR "lactobacillus" OR "bifidobacterium" OR "saccharomyces" OR "bacillus" OR "streptococcus thermophilus" OR "enterococcus" OR "lactococcus" OR "probiotic bacteria" OR "probiotic agent*") |
| #2 | TITLE-ABS-KEY ("irritable bowel syndrome" OR "IBS" OR "spastic colon" OR "irritable colon" OR "functional bowel disorder*" OR "mucous colitis" OR "irritable bowel" OR "spastic colitis" OR "IBS-D" OR "IBS-C" OR "IBS-M" OR "diarrhea predominant" OR "constipation predominant" OR "mixed type") |
| #3 | TITLE-ABS-KEY ("systematic review" OR "systematic reviews" OR "meta-analysis" OR "meta analysis" OR "metaanalysis" OR "umbrella review" OR "overview of reviews" OR "review of reviews" OR "scoping review" OR "narrative review" OR "pooled analysis") |
| #4 | #1 AND #2 AND #3 |
| Search strategy in Web of Science | |
| #1 | TS=("probiotic*" OR "lactobacillus" OR "bifidobacterium" OR "saccharomyces" OR "bacillus" OR "streptococcus thermophilus" OR "enterococcus" OR "lactococcus" OR "probiotic bacteria" OR "probiotic agent*") |
| #2 | TS=("irritable bowel syndrome" OR "IBS" OR "spastic colon" OR "irritable colon" OR "functional bowel disorder*" OR "mucous colitis" OR "irritable bowel" OR "spastic colitis" OR "IBS-D" OR "IBS-C" OR "IBS-M" OR "diarrhea predominant" OR "constipation predominant" OR "mixed type") |
| #3 | TS=("systematic review" OR "systematic reviews" OR "meta-analysis" OR "meta analysis" OR "umbrella review" OR "overview of reviews" OR "review of reviews" OR "scoping review" OR "narrative review") |
| #4 | #1 AND #2 AND #3 |
| Search strategy in EMBASE | |
| #1 | 'irritable colon'/exp OR 'irritable bowel syndrome':ti,ab,kw OR 'ibs':ti,ab,kw OR 'spastic colon':ti,ab,kw OR 'irritable colon':ti,ab,kw OR 'functional bowel disorder*':ti,ab,kw OR 'mucous colitis':ti,ab,kw OR 'irritable bowel':ti,ab,kw OR 'ibs-d':ti,ab,kw OR 'ibs-c':ti,ab,kw OR 'ibs-m':ti,ab,kw |
| #2 | ('irritable colon'/exp OR 'irritable bowel syndrome':ti,ab,kw OR 'ibs':ti,ab,kw OR 'spastic colon':ti,ab,kw OR 'irritable colon':ti,ab,kw OR 'functional bowel disorder*':ti,ab,kw OR 'mucous colitis':ti,ab,kw OR 'irritable bowel':ti,ab,kw OR 'ibs-d':ti,ab,kw OR 'ibs-c':ti,ab,kw OR 'ibs-m':ti,ab,kw) AND ('systematic review'/exp OR 'meta analysis'/exp OR 'systematic review':ti,ab,kw OR 'systematic reviews':ti,ab,kw OR 'meta-analysis':ti,ab,kw OR 'meta analysis':ti,ab,kw OR 'metaanalysis':ti,ab,kw OR 'umbrella review':ti,ab,kw OR 'overview of reviews':ti,ab,kw OR 'review of reviews':ti,ab,kw OR 'scoping review':ti,ab,kw OR 'narrative review':ti,ab,kw OR 'pooled analysis':ti,ab,kw) |
| #3 | 'systematic review'/exp OR 'meta analysis'/exp OR 'review'/exp OR 'systematic review':ti,ab,kw OR 'systematic reviews':ti,ab,kw OR 'meta-analysis':ti,ab,kw OR 'meta analysis':ti,ab,kw OR 'metaanalysis':ti,ab,kw OR 'umbrella review':ti,ab,kw OR 'overview of reviews':ti,ab,kw OR 'review of reviews':ti,ab,kw OR 'scoping review':ti,ab,kw OR 'narrative review':ti,ab,kw OR 'pooled analysis':ti,ab,kw |
| #4 | #1 AND #2 AND #3 |
| Search strategy in Cochrane Database of Systematic Reviews | |
| #1 | (MeSH descriptor: [Probiotics] explode all trees OR (probiotic* OR lactobacillus OR bifidobacterium OR saccharomyces OR bacillus OR "streptococcus thermophilus" OR enterococcus OR lactococcus):ti,ab,kw) |
| #2 | (MeSH descriptor: [Irritable Bowel Syndrome] explode all trees OR ("irritable bowel syndrome" OR IBS OR "spastic colon" OR "irritable colon" OR "functional bowel" OR "mucous colitis"):ti,ab,kw) |
| #3 | #1 AND #2 |
