# Supplementary material for: Probiotics in Irritable Bowel Syndrome: An Umbrella Review of 27 Systematic Reviews on Methodological Quality and Certainty of Evidence
Source: J Clin Med. 2026 Feb 25;15(5):1727. doi: 10.3390/jcm15051727 (PMC12985868; doi:10.3390/jcm15051727)
Supplement: Supplementary file 1 [file jcm-15-01727-s001.zip › Supplementary Material/Table S3.docx]

**Supplementary material Table 3.** Methodological characteristics of included systematic reviews.

| **First author, year** | **Review type** | **No. trials / participants** | **Diagnostic criteria** | **IBS subtype** | **Protocol registration** | **Conflicts of interest/funding** |
| --- | --- | --- | --- | --- | --- | --- |
| Yu Q-X, 2025 | SR with MA | 37 / 4,360 | Rome II-III | All | Not reported | No conflicts |
| Almabruk BA, 2024 | SR with MA | 23 / 3,288 | Mainly Rome III; some Rome II | All | Not reported | No conflicts |
| Wu Y, 2024 | SR with NMA | 54 / 6,732 | Rome I-IV, Manning | All | PROSPERO | No conflicts |
| Umeano L, 2024 | SR | 8 / (40-400 per study) | Rome III-IV | All | Not reported | No conflicts |
| Yang R, 2024 | SR with MA | 37 / 5,174 | Rome I-IV | All | Not reported | No conflicts |
| Chen M, 2023 | SR with MA (3 levels) | 72 / 8,581 | 93% with Rome criteria | All | Open Science Framework | No conflicts |
| Goodoory VC, 2023 | Updated SR with MA | 82 / 10,332 | Rome I-IV, Manning, physician opinion | All | Not reported | 2 authors with industry ties |
| Qing Q, 2023 | SR with MA | 7 / 883 | Rome II-III | All | Not reported | No conflicts |
| Xie P, 2023 | SR with NMA | 81 / 9,253 | Rome I-IV, Manning | All | PROSPERO | 45 RCTs with commercial funding |
| Konstantis G, 2023 | SR with MA | 6 / 970 | Rome IV only | All | protocols.io | No conflicts |
| Wang Y, 2022 | SR with MA | 10 / 943 | Rome II-III | IBS-D only | Research Registry | 7 trials with commercial funding |
| van der Geest AM, 2022 | SR with MA | 32 (14 probiotics) / 794-10,294 | Clinical opinion, Manning, Kruis, Rome I-IV | All | Not reported | 3 authors with industry ties |
| Shang X, 2022 | SR with MA | 10 / 757 | Rome I-IV | IBS-C only | Research Registry | No conflicts |
| Xie CR, 2022 | SR with NMA | 76 (65 probiotics) / 8,058 | Manning, Kruis, Rome I-IV | All | Not reported | No conflicts |
| Wen Y, 2020 | SR with MA | 17 / 1,469 | Rome I-III, clinical symptoms | IBS-C only | Not reported | No conflicts |
| Li B, 2020 | SR with MA | 59 / 6,721 | Rome I-III, Manning | All | Not reported | No conflicts |
| Niu HL, 2020 | SR with MA | 35 / 3,452 | Rome I-III, Kruis, physician opinion | All | Not reported | No conflicts |
| Sun JR, 2020 | SR with MA | 28 / 3,606 | Rome I-IV, clinical criterion | All | Not reported | No conflicts |
| Dale HF, 2019 | SR | 11 / ~1,600 | Rome III-IV | All | Not reported | No conflicts |
| Liang D, 2019 | SR with NMA | 14 / ~1,695 | Rome II-III | All | Not reported | No conflicts |
| Connell M, 2018 | SR with MA | 5 / 243 | Rome II-III | IBS-D only | Not reported | No conflicts |
| Ford AC, 2018 | SR with MA | 53 / 5,545 | Rome I-IV, Manning, Kruis | All | Not reported | 2 authors with industry ties |
| Yuan F, 2017 | SR with MA | 5 / 666 | Rome I-III | All | Not reported | No conflicts |
| Didari T, 2015 | SR with MA | 24 / 1,793 | Rome II-III, ICHPPC/WONCA | All | Not reported | No conflicts |
| Moayyedi P, 2010 | SR with MA | 19 / 1,650 | Rome I-III, Manning | All | Not reported | Funding and industry ties |
| Brenner DM, 2009 | SR | 16 / (12-362 per study) | Rome II, Manning | All | Not reported | No conflicts |
| Hoveyda N, 2009 | SR with MA | 12 / 1,134 | Rome I-II, Manning, clinical definition | All | Not reported | No conflicts |
