# Supplementary material for: Probiotics in Irritable Bowel Syndrome: An Umbrella Review of 27 Systematic Reviews on Methodological Quality and Certainty of Evidence
Source: J Clin Med. 2026 Feb 25;15(5):1727. doi: 10.3390/jcm15051727 (PMC12985868; doi:10.3390/jcm15051727)
Supplement: Supplementary file 1 [file jcm-15-01727-s001.zip › Supplementary Material/Table S4.docx]

**Supplementary Material Table 4.** Main findings of each study, sensitivity analyses, and reported adverse events

| **First author** | **Year** | **Main findings** | **Subgroup analyses and modifying factors** | **Adverse events** |
| --- | --- | --- | --- | --- |
| Yu Q-X | 2025 | **Global symptom improvement (37 RCTs):** 93.5% in combined group (2036/2177) vs. 73.8% in trimebutine alone (1612/2183).  OR (presenting improvement) = 5.09 (95%CI: 4.19–6.20; I² = 0%).  NNT ≈ 5 | **Global symptom improvement:**  Triple Bifidobacterium (22 studies): OR = 4.94 (95%CI: 3.78–6.46)  Compound Lactobacillus (4 studies): OR = 3.81 (95%CI: 2.13–6.82)  Quadruple Bifidobacterium (3 studies): OR = 4.75 (95%CI: 2.82–8.00).  Bacillus coagulans (3 studies): OR = 4.74 (95%CI: 2.34–9.62).  Bacillus subtilis (2 studies): OR = 10.44 (95%CI: 4.17–26.13).  Saccharomyces boulardii (2 studies): OR = 4.19 (95%CI: 1.71–10.29).  Low or no heterogeneity for all subgroups | Reported in 18 studies:  Trimebutine + probiotics: 38 cases (1.75%)  Trimebutine alone: 37 cases (1.69%), mainly: xerostomia, nausea, and dizziness. |
| Almabruk BA | 2024 | **Global symptom improvement:**  Majority of studies showed improvement (≥30% reduction in total severity or reported feeling "considerably/completely relieved" in ≥50% of treatment time).  **Abdominal pain:**  Significant pain reduction (20 RCTs): MD = –1.66 (95%CI –2.39 to –0.93; I² = 99.5%).  **Abdominal bloating:**  Significant score reduction (17 studies): MD = –2.13 (95%CI –3.96 to –0.30; I² = 99.7%).  **Quality of life:**  Improvement (13 RCTs): +8.77 points (95%CI +0.91 to +16.64; I² = 99.5%) | **Global symptom improvement**  Benefit with probiotics is more consistent in IBS-D; in IBS-C evidence is less robust; in mixed, benefits reported with specific strains.  **Abdominal pain**  By IBS subtype: benefit mainly in IBS-D; IBS-C less consistent; mixed with benefits in some trials.  **Abdominal bloating**  By IBS subtype: improvements noted more clearly in IBS-D.  **Quality of life (QoL)**  By IBS subtype: general improvement described, greater in IBS-D. | Infrequent and mild in most studies. |
| Wu Y | 2024 | **Global symptoms:**  Significant improvement (symptom reduction, 54 RCTs): SMD = –0.48 (95%CI –0.62 to –0.35; I² = 68.8%).  Failure to improve (54 RCTs): OR = 0.53 (95%CI 0.48–0.59; I² = 0%)  **Abdominal pain:**  Pain score (6 RCTs): SMD = –0.46 (95%CI –0.79 to –0.14; I² = 66.2%).  **Abdominal bloating:**  Bloating score (7 RCTs): SMD = not significant  **Quality of life (IBS-QoL):**  Lower symptom score (7 RCTs): SMD = –0.35 (95%CI –0.54 to –0.15; I² = 34.2%) | **Global symptoms**  Sensitivity (45 RCTs): SMD = −0.41 (−0.59 to −0.23; I²: not reported).  Bifidobacterium (4 RCTs): SMD = −0.48 (−0.89 to −0.07; I² ≈69.47%)  Lactobacillus (8 RCTs): SMD = −0.49 (−0.92 to −0.06; I² ≈ 53.7%)  Saccharomyces (4 RCTs): SMD = −0.42 (−0.78 to −0.06; I² ≈ 57.8%)  **Abdominal pain**  Bifidobacterium (3 RCTs): SMD= −0.51 (−0.89 to −0.14; I² ≈ 80%).  Lactobacillus and Saccharomyces: not significant  **Abdominal bloating**  Bifidobacterium (3 RCTs): SMD = −0.43 (−0.87 to 0.01; I² ≈85.64%).  Saccharomyces/Clostridium: Not significant.  **Quality of life**  Lactobacillus, Clostridium, E. coli: Not significant. | No serious adverse events observed; ~9.7% in intervention groups and ~8.3% in control (433 events in 5,031 subjects). |
| Umeano L | 2024 | **Global symptoms:**  7/8 trials show clinically relevant improvement; 5/8 with clear superiority vs placebo.  **Abdominal pain:**  Superiority vs placebo in 4 trials, intra-group improvements without superiority in 2; large effect in B. coagulans (2 studies)  **Abdominal bloating:**  Consistent reductions in studies explicitly measuring it (2 studies).  **Quality of life:**  Improvement in majority; superiority vs placebo reported in 3 studies | **Global symptoms:**  IBS-D: improvement in 2 studies. Multi-strain: consistent superiority (4 studies). Single-strain: heterogeneous effect (2 studies).  **Abdominal pain:**  IBS-D: significant reduction in 2. Multi-strain: improvement in 2 studies. Single-strain: 1 study superior to placebo (DDS-1/UABla-12) + B. coagulans (2 studies) with intra-group decreases.  **Abdominal bloating:**  IBS-D: improvement in 2. Single-strain: 2 studies with significant decrease. Multi-strain: 1 study with improvements.  **Quality of life:**  IBS-D: significant improvement in 3 studies. Multi-strain: more consistent pattern vs placebo. Single-strain: 1 with significant improvement. | Most common: mild gastrointestinal symptoms.  Rarely reported serious problems. |
| Yang R | 2024 | **Global symptom improvement:**  Response rate (20 RCTs): RR = 1.40 (95%CI: 1.18–1.66; I² = 71.7%)  Global symptom scores (18 RCTs): SMD = 0.4 (95%CI: 0.17–0.26; I² = 91.9%).  **Abdominal pain:**  Pain improvement (23 RCTs): SMD = 0.39 (95%CI: 0.12–0.65; I² > 84%).  **Abdominal bloating:**  Bloating improvement (18 RCTs): not significant (SMD = 0.112 (95%CI –0.008 to 0.231)  **Quality of life:**  Score improvement (13 RCTs): SMD = 0.29 (95%CI: 0.15–0.42; I² = 41.9%) | **Abdominal pain**  Multi-strain: SMD = 0.412 (0.112–0.711); I² and N not reported  Single-strain (not significant): SMD = 0.395 (–0.021 to 0.810); I² and N not reported  Dose ≥10¹⁰ CFU/day: SMD = 0.590 (0.050–1.129); I² and N not reported  Dose <10¹⁰ CFU/day (not significant): SMD = 0.276 (–0.015 to 0.566); I² and N not reported  **Abdominal bloating**  Duration <8 weeks: SMD = 0.197 (0.038–0.356); I² and N not reported  Duration ≥8 weeks (not significant): SMD = 0.014 (–0.158 to 0.186); I² and N not reported | Probiotics: 398/1835 (21.7%).  Placebo: 399/1680 (23.8%).  RR = 0.997 (95%CI: 0.85–1.18; p = 0.973) |
| Chen M | 2023 | **Global IBS symptoms:**  Symptom severity (63 RCTs): SMD = −0.55 (95%CI −0.76 to −0.34; I²= 96.3%).  **Abdominal pain:**  Pain score (48 studies): SMD = −0.89 (95%CI −1.29 to −0.50; I²= 98.4%).  **Abdominal bloating:**  Bloating sensation (N not reported) SMD = −0.77 (95%CI −1.20 to −0.35; I²= NR)  **Quality of life (QoL):**  Quality of life score (23 studies): SMD 0.99 (95%CI 0.45 to 1.54; I² = 98.0%). | **Global symptoms**  Treatment duration: <4 weeks: SMD = −1.00 (95%CI −1.37 to −0.63); 4–8 weeks: −0.56 (95%CI −0.88 to −0.24); 8 weeks: not significant.  Strains: Bifidobacterium: −0.77 (95%CI −1.42 to −0.12); Lactobacillus: −0.60 (95%CI −1.07 to −0.13); Saccharomyces: not significant.  **Abdominal pain**  Treatment duration: <4 weeks: SMD = −1.45 (95%CI −2.24 to −0.65); 4–8 weeks: −0.89 (95%CI −1.56 to −0.22); 8 weeks: not significant.  Strains: Bacillus: SMD = −2.23 (95%CI −4.31 to −0.14); Lactobacillus: −1.04 (95%CI −1.78 to −0.31); Saccharomyces: not significant  **Quality of life (QoL)**  Treatment duration: <4 weeks: SMD = +6.58 (95%CI +4.13 to +9.02); 4–8 weeks: +0.99 (95%CI +0.44 to +1.54); 8 weeks: not significant.  Strains: Bifidobacterium: +0.77 (95%CI −0.27 to +1.81); Saccharomyces: +6.45 (95%CI +4.36 to +8.55).  I² and N not reported in any of the items | Not reported |
| Goodoory VC | 2023 | **Global symptoms:**  Symptom persistence (32 RCTs)= RR 0.78 (95%CI: 0.71–0.87; I² = 71%)  **Abdominal pain:**  Persistent pain (32 RCTs): RR = 0.72 (95%CI: 0.64–0.82; I² = 72%).  **Abdominal bloating**  Persistent bloating (26 RCTs): RR = 0.75 (95%CI: 0.64–0.88; I² = 78%) | **Global symptom improvement**  LacClean Gold brand S RR = 0.59 (0.37–0.93); Duolac 7s RR = 0.62 (0.43–0.89); I² and N not reported.  Lactobacillus (16 studies): RR = 0.84 (0.72–0.98; I² = 69%).  L. plantarum 299V (5 studies): RR = 0.73 (0.59–0.92; I²= NR)  Bifidobacterium (5 studies): RR = 0.82 (0.67–1.02; I²= NR)  Bacillus (3 studies): RR = 0.44 (0.34–0.57; I²= NR)  Saccharomyces (2 studies): RR = 0.94 (0.80–1.11; I²: NR).  Escherichia (2 studies): RR = 0.86 (0.79–0.93; I²: NR).  IBS-D subtype (combinations, 13 studies): RR = 0.78 (0.67–0.92; I²: NR)  IBS-D (Lactobacillus): RR = 0.57 (0.36–0.89); I² and N not reported.  **Abdominal pain**  Lactobacillus (11 studies): RR = 0.59 (0.45–0.76; I² = 73%).  L. plantarum 299V (3 studies): not significant  Saccharomyces (9 studies): RR = 0.75 (0.57–0.99; I² = NR).  S. cerevisiae I-3856 (5 studies): RR = 0.64 (0.45–0.90; I²: NR).  S. boulardii (3 studies): not significant  Bifidobacterium (3 studies): RR = 0.78 (0.64–0.95; I²: NR).  Bacillus (3 studies): RR = 0.33 (0.23–0.47; I²: NR).  **Abdominal bloating:**  Lactobacillus (5 studies): not significant  Saccharomyces (5 studies): not significant  Bacillus (3 studies): RR = 0.41 (0.31–0.56; I²= NR). | Not significant (55 studies): RR = 1.05; (95%CI: 0.90–1.22; I² = 34%). |
| Qing Q | 2023 | **Abdominal pain**  Pain score (5 RCTs): SMD = −0.205 (95%CI: −0.347 to −0.063; I² = 28.7%).  **Abdominal bloating:**  Bloating score (4 RCTs): Not significant  **Quality of life:**  2/3 RCTs showed modest improvements | **Abdominal pain**  S. boulardii (3 RCTs): SMD = −0.316 (95%CI −0.611 to −0.020; I²: NR).  S. cerevisiae (2 RCTs): not significant  Sensitivity (5 RCTs after exclusions): SMD −0.205 (95%CI −0.347 to −0.063; I² = 28.7%).  **Abdominal bloating**  S. boulardii (3 RCTs) and S. cerevisiae (1 RCT): not significant  Sensitivity (4 RCTs after exclusions): not significant  **Quality of life (IBS-QOL)**  S. boulardii: improvements by domains in 2 RCTs.  S. cerevisiae: 1 RCT without changes. | No serious events observed; mild event rate ranged from 0–51.4% and was similar to control. |
| Xie P | 2023 | **Global symptom improvement (IBS-SSS)**  Lactobacillus acidophilus DDS-1 vs placebo (symptom presence, 18 RCTs): MD −77.70 (95%CI −101.72 to −53.68; I²=NR).  **Abdominal pain**  Bacillus coagulans MTCC 5856 vs placebo (pain score, 47 RCTs): SMD −41.80 (95%CI −61.59 to −22.00; I²=NR).  **Abdominal bloating:**  BL + LR mixture (B. longum + L. rhamnosus) vs placebo (bloating score, 39 RCTs): SMD −34.00 (95%CI −56.94 to −11.06; I²=NR).  **Quality of life (IBS-QOL):**  LP + LC + BL + ST + LA + X mixture vs placebo (score improvement, 9 RCTs): MD +15.35 (95%CI +4.45 to +26.26; I²=NR). | **Global symptom improvement (IBS-SSS)**  BL + LR: MD = −80.99 (95%CI: −130.73 to −31.26).  LC + LP + BAL: MD = −76.42 (95%CI: −114.90 to −37.95).  LP + LC + BL + ST + LA + X: MD = −63.96 (95%CI: −78.66 to −49.26).  B. lactis UABla-12: MD = −48.80 (95%CI: −73.00 to −24.60).  EF + LA + X: MD = −35.00 (95%CI: −60.44 to −9.56).  B. bifidum MIMBb75: MD = −29.83 (95%CI: −48.24 to −11.42).  C. butyricum CGMCC0313.1: MD = −21.38 (95%CI: −40.47 to −2.29).  BAL + LA + BB + X: MD = −18.86 (95%CI: −25.88 to −11.85).  **Abdominal pain**  B. coagulans Unique IS2: SMD = −32.00 (95%CI: −45.35 to −18.65).  L. gasseri BNR17: SMD = −36.10 (95%CI: −64.53 to −7.67).  L. plantarum Apsulloc 331261: SMD = −26.59 (95%CI: −47.07 to −6.11).  L. acidophilus DDS-1: SMD = −19.53 (95%CI: −33.49 to −5.57).  LPA + LS + LP: SMD = −20.00 (95%CI: −35.97 to −4.03).  S. cerevisiae CNCM I-3856: SMD = −15.24 (95%CI: −24.62 to −5.87).  VSL#3: SMD = −12.93 (95%CI: −25.59 to −0.26).  EF + LA + X: SMD = −11.37 (95%CI: −21.68 to −1.06).  LA + ST + X: SMD = −8.14 (95%CI: −15.54 to −0.75).  **Abdominal bloating**  BL + LR: SMD = −34.00 (95%CI: −56.94 to −11.06).  L. plantarum CCFM8610: SMD = −19.92 (95%CI: −34.91 to −4.91).  L. plantarum 299v: SMD = −14.79 (95%CI: −29.11 to −0.48).  VSL#3: SMD = −13.71 (95%CI: −22.12 to −5.30).  B. bifidum MIMBb75: SMD = −11.83 (95%CI: −22.93 to −0.74).  **Quality of life (IBS-QOL)**  LP + LC + BL + ST + LA + X: MD = +15.35 (95%CI: +4.45 to +26.26).  C. butyricum CGMCC0313.1: MD = +4.07 (95%CI: +0.50 to +7.65).  I² and N not reported in any of the items | Total adverse events (23 RCTs):  11.62% in probiotic group (452/3891).  10.61% in placebo group (379/3572).  OR = 0.92 (95%CI 0.73–1.15; I²=NR)  Serious adverse events:  0.15% in probiotics (6/4081).  0.22% in placebo (8/3679). |
| Konstantis G | 2023 | **Global symptom improvement**  WMD= Not significant  **Abdominal pain:**  Pain score (6 studies): SMD = −0.94 (95%CI: −1.53 to −0.35; I² = 92.2%)  **Abdominal bloating:**  Bloating score (6 studies): SMD = −0.28 (95%CI: −0.47 to −0.09; I² = 36.1%)  **Quality of life:**  Not significant | **Global symptom improvement (IBS-SSS)**  Without FODMAP (2 RCTs): WMD = −75.5 (95%CI −108.7 to −42.3; I² = 49.7%)  Sensitivity (Only low risk of bias; 2 RCTs): Not significant  **Abdominal pain**  With FODMAP (2 RCTs): Not significant  Without FODMAP (4 RCTs): SMD = −1.27 (95%CI −1.87 to −0.67; I² = 89.4%).  Only low risk of bias (2 RCTs): Not significant  **Abdominal bloating**  With FODMAP (2 RCTs): Not significant  Without FODMAP (4 RCTs): SMD = −0.41 (95%CI −0.75 to −0.08; I² = 69.7%).  Only low risk of bias (2 RCTs): SMD = −0.20 (95%CI −0.39 to −0.01; I² = 41.1%).  **Quality of life (QoL)**  VAS-QoL instrument (2 RCTs): SMD = −1.51 (95%CI −2.04 to −1.01; I² = 0%).  IBS-QoL instrument (2 RCTs): SMD = −0.19 (95%CI −0.34 to −0.04; I² = 0%).  Only low risk of bias (k = 3): SMD = −0.18 (95%CI −0.32 to −0.04); I² = 0%. | No serious events, mild events were similar to placebo |
| Wang Y | 2022 | **Global IBS-D symptoms:**  Symptom severity score (8 RCTs): SMD = −0.55 (95%CI −0.83 to −0.27; I² ≈ 66%).  **Abdominal pain:**  Pain score (8 RCTs): SMD = −0.43 (95%CI −0.57 to −0.29; I² ≈ moderate),  **Abdominal bloating:**  Bloating score (8 RCTs): SMD = −0.45 (95%CI −0.81 to −0.09; I² = 76%)  **Quality of life:**  Not significant | **Global symptoms:**  Single-strain (2 RCTs): SMD = −0.25 (95%CI −0.49 to −0.01; I² = 0%).  Multi-strain (6 RCTs): SMD = −0.68 (95%CI −0.98 to −0.42; I² = 72%).  **Abdominal pain:**  Single-strain (3 RCTs): not significant.  Multi-strain (5 RCTs): SMD ≈ −0.49 (95%CI −0.70 to −0.28; I² = 55%).  **Abdominal bloating:**  Multi-strain (5 RCTs): SMD = −0.67 (95%CI −1.13 to −0.21; I² = 69%).  Single-strain (3 RCTs): not significant.  **Quality of life:**  Single-strain (2 RCTs): SMD = 0.37 (95%CI 0.13 to 0.61; I² = 0%).  Multi-strain (5 RCTs): not significant. | 10.6% (31/293) probiotics vs 9.2% (27/294) placebo  No significant difference between probiotics and placebo (RR = 1.10; 95%CI: 0.72–1.69; I²: 0%) |
| van der Geest AM | 2022 | **Global symptoms:**  Symptom persistence (11 RCTs): RR = 0.68 (95%CI: 0.51–0.92; I² = 82%).  **Abdominal pain:**  Pain score (10 RCTs): SMD = –0.35 (95%CI: –0.56 to –0.14, I² = 77%).  **Abdominal bloating:**  Bloating score (4 RCTs): not significant | Not reported | Favorable overall profile: no serious AEs attributable to probiotics described.  AEs were mainly mild and transient gastrointestinal (e.g., gas, bloating). |
| Shang X | 2022 | **Abdominal pain:**  Pain score (4 RCTs): Not significant  **Abdominal bloating:**  Bloating score (2 RCTs): Not significant  **Quality of life:**  Bloating score (3 RCTs): Not significant | **Abdominal pain**  8 weeks (≈2 RCTs): SMD = −1.28 (95%CI −2.26 to −0.29; I2:)  6 weeks (1 RCT) and 12 weeks (1 RCT): not significant.  Sensitivity analysis (Excluding Gayathri 2019, 3 RCTs): SMD = −0.20 (95%CI −0.38 to −0.01; I² = 0%).  **Abdominal bloating**  12 weeks (1 RCT): SMD = −0.31 (95%CI −0.61 to −0.02)  **Quality of life**  8 weeks (1 RCT): SMD = −8.06 (95%CI −8.92 to −7.21)  6 weeks (1 RCT) and 12 weeks (1 RCT): not significant. | No significant differences vs placebo (4 RCTs): OR = 1.57 (95%CI: 0.87–2.82; I²= NR).  Reported events: abdominal pain, anal irritation, gastroesophageal reflux, constipation, and cramps. |
| Xie CR | 2022 | **Global symptom improvement:**  Symptom improvement (9 RCTs): RR = 1.50 (95%CI 1.10–2.05; I² = 71.1%)  **Abdominal pain:**  Pain score (11 RCTs): SMD = −1.51 (95%CI −2.18 to −0.85; I² = 96.2%) | **Global symptom improvement**  Lactobacillus (8 RCTs): RR 1.74 (95%CI: 1.22–2.48; I² = NR).  Bifidobacterium (4 RCTs): RR 1.76 (95%CI: 1.01–3.07; I² = NR).  Bacillus (1 RCT): RR 5.67 (95%CI: 1.85–17.40; I²= NR).  IBS-D subgroup: "no significant differences".  **Abdominal pain / global score**  Lactobacillus (10 RCTs): SMD −0.90 (95%CI −1.54 to −0.27; I²= NR)  Bacillus (4 RCTs): SMD −2.31 (95%CI −3.91 to −0.71; I²=NR)  BL (6 RCTs): SMD −1.28 (95%CI −2.19 to −0.36; I² = NR)  IBS-D subgroup: "no significant differences". | Probiotics: 16.4% (315/1,921).  Placebo: 14.3% (230/1,607); Frequent symptoms: mild gastrointestinal; isolated cases: headache, urticaria, fatigue. |
| Wen Y | 2020 | **Global symptom improvement:**  Relief satisfaction (3 RCTs): not significant.  Failure to respond to treatment (2 RCTs): RR =not significant  **Abdominal bloating:**  Bloating score (4 RCTs): SMD = −0.77 (95%CI: −1.46 to −0.07; I² = NR). | Not reported | No serious adverse events. 6 RCTs had no events; 1 RCT reported mild events. High tolerability (91% vs 80% in one RCT) and adherence >95% in 2 RCTs. |
| Li B | 2020 | **Global symptom improvement:**  Symptom improvement (35 RCTs): RR = 1.52 (95%CI 1.32–1.76; I² = 71%).  Total symptom score (29 RCTs): SMD = - 0.18 (95%CI −0.30 to −0.06; I² = 65%).  **Abdominal pain:**  Abdominal pain score (38 RCTs): SMD = −0.22 (95%CI −0.33 to −0.11; I² = 70%).  **Abdominal bloating:**  Abdominal bloating score (29 RCTs): SMD = −0.13 (95%CI −0.24 to −0.03; I² = 54%). | **Global symptoms**  Duration <8 weeks (18 RCTs): RR = 1.55 (95%CI: 1.27–1.89; I² = 74%).  Duration ≥8 weeks (17 RCTs): RR = 1.52 (95%CI: 1.23–1.88; I² = 69%).  High doses (≥10¹⁰ CFU/day, 21 RCTs): RR = 1.51 (95%CI: 1.20–1.91; I² = 77%).  Low doses (<10¹⁰ CFU/day, 15 RCTs): RR = 1.56 (95%CI: 1.33–1.83; I² = 54%).  Single-strain (15 RCTs): RR = 1.76 (95%CI: 1.37–2.25; I² = 69%).  Multi-strain (21 RCTs): RR = 1.39 (95%CI: 1.18–1.65; I² = 60%).  Low risk of bias trials (19 RCTs): RR = 1.59 (95%CI: 1.25–2.04, I² = NR).  Duration <8 weeks (11 RCTs): SMD = No significant differences  Single-strain (14 RCTs): SMD = −0.06 (−0.16 to 0.14; I² = 33%).  **Abdominal pain:**  High doses (24 RCTs): SMD = −0.14 (95%CI: −0.26 to −0.01; I² = 39%).  **Abdominal bloating:**  Duration <8 weeks (19 RCTs): SMD = −0.13 (95%CI: −0.27 to −0.01; I² = 47%). | 40 studies provided adverse event information, 14 trials indicated zero events.  No significant differences: RR = 1.07 (95%CI: 0.92–1.24; I² = 0) |
| Niu HL | 2020 | **Global symptom improvement:**  Symptom persistence (23 RCTs): RR 0.79 (95%CI 0.70–0.89; I² = 72%).  **Abdominal pain:**  Pain score (24 RCTs): SMD −0.25 (95%CI −0.36 to −0.14, I² = 27%)  **Abdominal bloating:**  Bloating score (17 RCTs): SMD −0.15 (95%CI −0.27 to −0.03, I² = 16%) | **Global symptoms:**  Multi-strain (12 RCTs): RR 0.81 (0.67–0.98).  Lactobacillus (6 RCTs), Bifidobacterium (2 RCTs): Not significant.  Escherichia (2 RCTs): RR 0.86 (0.79–0.93).  Low risk of bias (16 RCTs): RR 0.77 (0.67–0.89).  High risk of bias (7 RCTs): Not significant.  Duration ≤4 wk (10 RCTs): RR 0.75 (0.61–0.93).  Duration >4 wk (13 RCTs): RR 0.81 (0.70–0.94).  **Abdominal pain:**  Multi-strain (15 RCTs): SMD −0.24 (−0.37 to −0.12).  Lactobacillus (6 RCTs), Bifidobacterium (3 RCTs), Saccharomyces (1 RCT): SMD = not significant  Low risk of bias (17 RCTs): SMD −0.27 (−0.40 to −0.15);  High risk of bias (7 RCTs): SMD: not significant.  Duration ≤4 wk (11 RCTs): SMD −0.32 (−0.49 to −0.14).  Duration >4 wk (13 RCTs): SMD −0.16 (−0.31 to −0.01).  **Abdominal bloating:**  Multi-strain (8 RCTs): SMD: not significant  Bifidobacterium (4 RCTs), Lactobacillus (3 RCTs), Saccharomyces (2 RCTs): SMD = not significant.  Low risk of bias (13 RCTs): SMD −0.19 (−0.32 to −0.06).  High risk of bias (4 RCTs): SMD = not significant.  Duration ≤4 wk (7 RCTs), >4 wk (10 RCTs): not significant.  I²: not reported in any subgroups | 201/1,215 (16.5%) in probiotics vs. 164/1,192 (13.8%) in placebo.  Higher risk in probiotics  RR = 1.21; 95%CI: 1.02–1.44. |
| Sun JR | 2020 | **Global symptom improvement:**  Global symptom improvement (22 RCTs): RR = 1.50 (95%CI: 1.23–1.83; I² = 68%).  Number needed to treat (NNT): 5 (95%CI: 3–8.7).  **Global symptoms or abdominal pain:**  Symptom and pain score (18 RCTs): SMD = –0.31 (95%CI: –0.45 to –0.17; I² = 66%).  **Abdominal bloating:**  Bloating score (15 RCTs): SMD = –0.20 (95%CI –0.38 to –0.01, I² = 76%).  **Quality of life:**  Quality score (5 RCTs): SMD = no significant benefit. | **Global symptom improvement**  Combination (12 RCTs): RR=1.47 (95%CI: 1.13–1.92, I²=68%)  NNT=6 (4.5–8.7).  Lactobacillus (5 RCTs); Bifidobacterium (2 RCTs), E. coli (2 RCTs); Saccharomyces (1 RCT): Not significant  **Global symptoms or abdominal pain**  Combination (12 RCTs): SMD −0.35 (95%CI: −0.57 to −0.13, I²=68%).  Lactobacillus (3 RCTs): not significant.  Bifidobacterium (3 RCTs): not significant | 323/923 (35%) in probiotics and 245/731 (33.5%) in placebo group.  Relative risk (RR) = 1.05 (95%CI: 0.85–1.31), no significant differences.  Most common adverse events: abdominal pain, diarrhea, constipation, nausea, skin reactions. |
| Dale HF | 2019 | **Global symptom improvement:**  7 RCTs show probiotic benefit vs placebo; 3 show no benefit; 1 did not clearly evaluate this outcome.  **Abdominal pain:**  5 RCTs show pain reduction with probiotics; 2 show no difference; 4 did not evaluate pain as specific outcome.  **Abdominal bloating:**  5 RCTs report decreased bloating; 2 show no difference; 4 did not evaluate bloating as specific outcome.  **Quality of life:**  3 RCTs show QoL improvement; 3 show no difference; 5 did not evaluate QoL. | **Global symptom improvement**  Multi-strain: 6/8 RCTs with global benefit; 2/8 without significant benefit.  Single-strain: 1/3 RCTs with global benefit; 2/3 without significant benefit.  IBS-D: 2/3 RCTs with global improvement; 1/3 without improvement and even negative signal.  IBS-C: 1 RCT with global improvement.  1 RCT found greater improvement in males; 2 RCTs no differences.  **Abdominal pain**  Multi-strain: 3 RCTs reported pain reduction.  Single-strain (Bacillus coagulans): 1 RCT with marked pain reduction.  Single-strain (Saccharomyces): 1 RCT no significant difference.  IBS-D: 2 RCTs with positive results; 1 study no significant differences.  IBS-C: 1 RCT with positive result.  **Abdominal bloating**  Multi-strain: improvements reported 2 RCTs; 1 RCT no differences.  Single-strain: Pineton (S. cerevisiae) no difference vs placebo.  **Quality of life (QoL)**  Multi-strain: 2 RCTs reported QoL improvement; 1 RCT found no difference.  Single-strain: 1 RCT (B. coagulans) improved QoL. | Probiotics were safe and well tolerated in all studies.  No serious adverse effects reported.  Described AEs were mild and similar to placebo, mainly gastrointestinal symptoms (bloating, gas, digestive discomfort). |
| Liang D | 2019 | **Global symptom improvement:**  Symptom relief satisfaction (13 RCTs): RR 1.27 (95%CI 1.13–1.44), I² = 34%. | **Global symptom improvement**  L+B: RR 1.39 (95%CI 1.19–1.61, I² = 49%).  Lactobacillus only (L): RR 1.02 (95%CI 0.75–1.38, I² = 0%).  Bifidobacterium only (B): RR 1.26 (95%CI 0.98–1.62, I² = 23%).  Low dose (10⁹–10¹⁰ cfu/day): RR 2.08 (95%CI 1.59–2.71, I² = 0%).  High dose (>10¹⁰ cfu/day): RR 1.09 (95%CI 0.86–1.37, I² = 0%).  **Network meta-analysis**  DUO vs placebo: RR 7.46 (95%CI 2.00–32.23, I² = N/A).  Pro vs placebo: RR 7.16 (95%CI 1.72–29.89, I² = N/A).  DUO vs F19: RR 6.37 (95%CI 1.34–32.59, I² = N/A).  DUO vs Bif: RR 6.66 (95%CI 1.08–47.34, I² = N/A).  F19 vs Pro: RR 0.16 (95%CI 0.03–0.88, I² = N/A).  Number of studies not reported in any subgroups | 12/14 RCTs reported adverse events, most infrequent, no serious AEs attributable to probiotics; mild and transient gastrointestinal discomfort predominates. |
| Connell M | 2018 | **Global symptom improvement:**  Symptom relief satisfaction (3 RCTs): RR =not significant  **Abdominal pain**  Pain intensity score reduction (5 trials): SMD = not significant  **Abdominal bloating:**  Pain score improvement (5 RCTs): SMD: not significant  **Quality of life (QOL)**  Quality of life score (3 trials): SMD = No significant improvement | **Global symptoms**  Sensitivity (excluding trial with 7-day response definition, 2 RCTs): RR = not significant  **Abdominal pain**  Sensitivity (excluding study with imputed SD, 4 RCTs): SMD = not significant  **Abdominal bloating**  Sensitivity (excluding study with imputed SD, 4 RCTs): SMD = not significant  **Quality of life**  Sensitivity (excluding study with imputed SD, 2 RCTs): SMD = not significant. | Reported only in 1 RCT: 3.8% VSL#3 vs 7.8%. |
| Ford AC | 2018 | **Global symptom improvement:**  Symptom persistence (21 RCTs): RR 0.79 (95%CI 0.68–0.91; I²=72%)  NNT ≈ 7 (95%CI 5–19).  **Abdominal pain:**  Pain score (14 RCTs): SMD −0.31 (95%CI −0.44 to −0.17; I²=24%).  **Abdominal bloating:**  Bloating score (24 RCTs): SMD = −0.135 (95%CI −0.34 to −0.01, I² = 76%). | **Global symptom improvement:**  L. paracasei F19 + L. acidophilus La5 + B. lactis Bb12 (3 RCTs): not significant  LacClean Gold (2 RCTs): RR 0.59 (95%CI: 0.37–0.93; I²=NR).  7-strain combination (2 RCTs): RR 0.48 (95%CI: 0.24–0.94; I²=NR).  L. plantarum DSM 9843 (3 RCTs): RR 0.67 (95%CI: 0.51–0.87; I²=63%).  Escherichia (2 RCTs): RR 0.86 (95%CI: 0.79–0.93; I²=NR).  Lactobacillus (8 RCTs); Bifidobacterium (3 RCTs); Saccharomyces cerevisiae (2 RCTs); VSL#3 (2 RCTs): not significant  **Abdominal pain:**  VSL#3 (4 RCTs); L. paracasei F19 + L. acidophilus La5 + B. lactis Bb12 (3 RCTs); B. lactis DN-173010 + S. thermophilus + L. bulgaricus (2 RCTs); Lactobacillus (8 RCTs); L. plantarum DSM 9843 (3 RCTs); Bifidobacterium (3 RCTs); B. infantis 35624 (2 RCTs): SMD = not significant  **Abdominal bloating:**  Bifidobacterium / Lactobacillus / Saccharomyces (separately): not significant | 19.4% (433/2228) in probiotic group.  17.0% (332/1955) in placebo group.  No significant difference (36 RCTs) = RR = 1.09 (95%CI: 0.91–1.29; I² = 36%), |
| Yuan F | 2017 | **Abdominal pain**  B. infantis (any formulation; 5 RCTs): SMD = not significant  **Abdominal bloating**  B. infantis (any formulation: 5 RCTs): SMD = 0.21 (95%CI 0.07 to 0.35; I²<50%) | **Abdominal pain**  B. infantis alone (3 RCTs): SMD = not significant  Combinations with B. infantis (2 RCTs): SMD = 0.22 (95%CI 0.03–0.41; I²<50%)  **Abdominal bloating**  B. infantis alone (3 RCTs): SMD = not significant  Combinations with B. infantis (2 RCTs): SMD = 0.30 (95%CI 0.04–0.56, I²<50%) | No serious adverse effects observed; symptoms were mild and in some cases even more frequent in placebo. |
| Didari T | 2015 | **Global symptom improvement**  Responders (2 RCTs): RR = 2.43 (95%CI 1.13–5.21; I²: NR).  Adequate symptom improvement (6 RCTs): RR = 2.14 (95%CI 1.08–4.26; I²: NR).  **Abdominal pain**  Responders (2 RCTs): RR = 1.96 (95%CI 1.14–3.36; I²: NR).  Pain scores (2 RCTs): SMD = not significant  **Abdominal bloating**  Bloating score (2 RCTs): SMD = not significant.  **Quality of life**  Narrative results (3/5 RCTs show improvement; others do not (2/5)) | **Global symptom improvement**  Time (1 RCT): 4–5, 13–14, 20 weeks favoring probiotics.  IBS subtype (1 RCT): no difference between subtypes.  **Abdominal pain**  IBS subtype (IBS-C/IBS-Mixed, 1 RCT): RR = not significant  VAS 0–100 (1 RCT): Greater reduction with probiotics.  **Abdominal bloating**  VAS 0–100 (1 RCT): No benefit. | Probiotics are considered safe and well tolerated in IBS patients. Adverse effects were mild, with no serious complications reported, and withdrawals due to adverse effects were few and comparable to placebo group. |
| Moayyedi P | 2010 | **Global symptom improvement**  Symptom persistence (10 RCTs): RR = 0.71 (95%CI 0.57–0.88; I²=68%)  NNT≈4 (95%CI 3–12.5).  Total symptom score (15 RCTs): SMD −0.34 (95%CI −0.60 to −0.07; I²=79%)  **Abdominal pain**  Pain scores (10 RCTs): SMD = −0.51 (95%CI −0.91 to −0.09; I²=85%).  **Abdominal bloating**  Bloating score (8 RCTs): SMD = not significant | **Global symptom improvement**  Dichotomous: "symptom persistence"  Jadad quality ≥4 (6 RCTs): not significant  Jadad quality <4 (4 RCTs): RR 0.52 (0.35–0.77; I²=NR).  Continuous: "total symptom score"  Sensitivity (excluding outlier, 14 RCTs): SMD = −0.18 (95%CI: −0.29 to −0.06; I²=0%)  Jadad ≥4 (excluding outlier, 9 RCTs): SMD = −0.19 (95%CI: −0.31 to −0.03; I²=NR).  Jadad <4 (excluding outlier, 5 RCTs): SMD = not significant  **Abdominal pain**  Sensitivity (excluding outliers, 9 RCTs): SMD −0.22 (95%CI: −0.37 to −0.06; I²=0%). | No significant differences = RR: 0.93 (95%CI: 0.64–1.36), 6 RCTs reported no adverse events. |
| Brenner DM | 2009 | **Global symptom improvement:**  B. infantis 35624: 2/2 RCTs show probiotic benefit vs placebo.  **Abdominal pain:**  B. infantis 35624: 2/2 RCTs show pain reduction with probiotics.  **Abdominal bloating:**  B. infantis 35624: 2/2 RCTs report decreased bloating.  **Quality of life:**  Lactobacillus reuteri ATCC 55730: 1/1 shows no difference. | **Global symptom improvement**  Best signal in L. plantarum 299v (small parallel RCT) and Lacteol Fort (crossover, with reservations); multi-strain (Kajander) shows late and fragile effect; VSL#3 does not consolidate globally.  **Abdominal pain:**  L. plantarum 299v suggests early but fragile benefit; VSL#3 and other mixtures → neutral.  **Abdominal bloating:**  VSL#3 and DN-173010 provide improvements (VSL#3 in bloating/flatulence; DN-173010 at 3 weeks without sustaining at 6); L. plantarum 299v is variable.  **Quality of life:**  L. reuteri (IBS-QOL) negative; in the rest, domains may improve but not validated global QoL. | Most studies did not report relevant adverse effects.  Symptoms were mild gastrointestinal (nausea, dyspepsia, flatulence, headache).  Some reported isolated serious events (angina, chest pain, epistaxis), without association with probiotic. |
| Hoveyda N | 2009 | **Global symptom improvement:**  Symptom improvement (6 RCTs): OR = 1.59 (95%CI 1.19–2.13; I² = 35%).  **Abdominal pain:**  Pain improvement (5 RCTs): OR = 3.34 (95%CI 1.99–5.61; I² = 1%).  **Abdominal bloating:**  Bloating improvement (4 RCTs): OR = 1.75 (95%CI 1.03–2.96; I² = 0%).  **Quality of life:**  Could not be meta-analyzed: Heterogeneous and inconclusive results. | **Global symptom improvement:**  Sensitivity (excluding lower quality studies, 3 RCTs): OR 1.62 (95%CI 1.06–2.48; I²=20%).  **Abdominal pain**  Sensitivity: Excluding 1 RCT did not change finding for pain (remained significant); numerical values not shown.  **Abdominal bloating**  Sensitivity: Excluding 1 RCT, the effect for bloating ceased to be significant; numerical values not shown. | 6 RCTs: no adverse events reported.  When there were AEs: mild and comparable to placebo: dyspepsia/headache/nausea, 1 RCT had 17 withdrawals due to AEs, no difference between groups. |
