# Supplementary material for: Probiotics in Irritable Bowel Syndrome: An Umbrella Review of 27 Systematic Reviews on Methodological Quality and Certainty of Evidence
Source: J Clin Med. 2026 Feb 25;15(5):1727. doi: 10.3390/jcm15051727 (PMC12985868; doi:10.3390/jcm15051727)
Supplement: Supplementary file 1 [file jcm-15-01727-s001.zip › Supplementary Material/Table S5.docx]

**Supplementary Material Table 5:** Downgrading of certainty of evidence according to GRADE domains (risk of bias, inconsistency, indirectness, imprecision, and publication bias) for the 92 outcomes evaluated in the 27 systematic reviews

| **Author (year)** | **Outcome** | **Risk of bias** | **Inconsistency** | **Indirectness** | **Imprecision** | **Publication bias** | **GRADE result** |
| --- | --- | --- | --- | --- | --- | --- | --- |
| Yu (2025) | General symptoms | Very serious (−2) | Not serious (0) | Serious (−1) | Not serious (0) | Serious (−1) | VERY LOW ⊕⊖⊖⊖ |
| Almabruk (2024) | General symptoms | Serious (−1) | Very serious (−2) | Serious (−1) | Serious (−1) | Serious (−1) | VERY LOW ⊕⊖⊖⊖ |
|  | Abdominal pain | Serious (−1) | Very serious (−2) | Serious (−1) | Serious (−1) | Serious (−1) | VERY LOW ⊕⊖⊖⊖ |
|  | Abdominal bloating | Serious (−1) | Very serious (−2) | Serious (−1) | Serious (−1) | Serious (−1) | VERY LOW ⊕⊖⊖⊖ |
|  | Quality of life | Serious (−1) | Very serious (−2) | Serious (−1) | Serious (−1) | Serious (−1) | VERY LOW ⊕⊖⊖⊖ |
| Wu (2024) | Abdominal pain | Not serious (0) | Serious (−1) | Not serious (0) | Serious (−1) | Serious (−1) | VERY LOW ⊕⊖⊖⊖ |
|  | Abdominal bloating | Not serious (0) | Serious (−1) | Not serious (0) | Serious (−1) | Unclear (0) | LOW ⊕⊕⊖⊖ |
| Umeano (2024) | General symptoms | Not serious (0) | Serious (−1) | Not serious (0) | Serious (−1) | Unclear (0) | LOW ⊕⊕⊖⊖ |
|  | Abdominal pain | Not serious (0) | Serious (−1) | Serious (−1) | Serious (−1) | Unclear (0) | VERY LOW ⊕⊖⊖⊖ |
|  | Abdominal bloating | Not serious (0) | Serious (−1) | Serious (−1) | Serious (−1) | Unclear (0) | VERY LOW ⊕⊖⊖⊖ |
|  | Quality of life | Not serious (0) | Serious (−1) | Not serious (0) | Serious (−1) | Unclear (0) | LOW ⊕⊕⊖⊖ |
| Yang (2024) | General symptoms (continuous) | Not serious (0) | Serious (−2) | Not serious (0) | Not serious (0) | Not serious (0) | LOW ⊕⊕⊖⊖ |
|  | Abdominal pain | Not serious (0) | Serious (−2) | Not serious (0) | Not serious (0) | Unclear (0) | LOW ⊕⊕⊖⊖ |
|  | Abdominal bloating | Not serious (0) | Serious (−1) | Not serious (0) | Serious (−1) | Unclear (0) | LOW ⊕⊕⊖⊖ |
| Chen (2023) | General symptoms | Serious (−1) | Serious (−1) | Not serious (0) | Not serious (0) | Unclear (0) | LOW ⊕⊕⊖⊖ |
|  | Abdominal pain | Serious (−1) | Serious (−1) | Not serious (0) | Not serious (0) | Not serious (0) | LOW ⊕⊕⊖⊖ |
|  | Abdominal bloating | Serious (−1) | Serious (−1) | Not serious (0) | Not serious (0) | Unclear (0) | LOW ⊕⊕⊖⊖ |
|  | Quality of life | Serious (−1) | Serious (−1) | Not serious (0) | Not serious (0) | Serious (−1) | VERY LOW ⊕⊖⊖⊖ |
| Goodoory (2023) | General symptoms | Serious (−1) | Serious (−1) | Not serious (0) | Not serious (0) | Serious (−1) | VERY LOW ⊕⊖⊖⊖ |
|  | Abdominal pain | Serious (−1) | Serious (−1) | Not serious (0) | Not serious (0) | Serious (−1) | VERY LOW ⊕⊖⊖⊖ |
|  | Abdominal bloating | Serious (−1) | Serious (−1) | Not serious (0) | Not serious (0) | Serious (−1) | VERY LOW ⊕⊖⊖⊖ |
| Qing (2023) | Quality of life | Not serious (0) | Serious (−1) | Not serious (0) | Serious (−1) | Unclear (0) | LOW ⊕⊕⊖⊖ |
| Konstantis (2023) | General symptoms | Serious (−1) | Serious (−1) | Not serious (0) | Serious (−1) | Unclear (0) | VERY LOW ⊕⊖⊖⊖ |
|  | Abdominal pain | Serious (−1) | Serious (−1) | Not serious (0) | Not serious (0) | Unclear (0) | LOW ⊕⊕⊖⊖ |
|  | Quality of life | Serious (−1) | Serious (−1) | Not serious (0) | Not serious (0) | Unclear (0) | VERY LOW ⊕⊖⊖⊖ |
| Wang (2022) | General symptoms | Very serious (−2) | Serious (−1) | Not serious (0) | Serious (−1) | Unclear (0) | VERY LOW ⊕⊖⊖⊖ |
|  | Abdominal pain | Very serious (−2) | Not serious (0) | Not serious (0) | Serious (−1) | Unclear (0) | VERY LOW ⊕⊖⊖⊖ |
|  | Abdominal bloating | Very serious (−2) | Serious (−1) | Not serious (0) | Not serious (0) | Unclear (0) | VERY LOW ⊕⊖⊖⊖ |
|  | Quality of life | Very serious (−2) | Serious (−1) | Not serious (0) | Serious (−1) | Unclear (0) | VERY LOW ⊕⊖⊖⊖ |
| Van der Geest (2022) | General symptoms | Not serious (0) | Serious (−1) | Not serious (0) | Not serious (0) | Serious (−1) | LOW ⊕⊕⊖⊖ |
|  | Abdominal bloating | Not serious (0) | Serious (−1) | Not serious (0) | Serious (−1) | Unclear (0) | LOW ⊕⊕⊖⊖ |
| Shang (2022) | Abdominal pain | Serious (−1) | Serious (−1) | Not serious (0) | Not serious (0) | Unclear (0) | LOW ⊕⊕⊖⊖ |
|  | Abdominal bloating | Serious (−1) | Serious (−1) | Not serious (0) | Not serious (0) | Unclear (0) | LOW ⊕⊕⊖⊖ |
|  | Quality of life | Serious (−1) | Very serious (−2) | Not serious (0) | Serious (−1) | Unclear (0) | VERY LOW ⊕⊖⊖⊖ |
| Xie (2022) | Abdominal pain | Not serious (0) | Serious (−1) | Serious (−1) | Not serious (0) | Unclear (0) | LOW ⊕⊕⊖⊖ |
| Wen (2020) | General symptoms | Serious (−1) | Not serious (0) | Not serious (0) | Serious (−1) | Unclear (0) | LOW ⊕⊕⊖⊖ |
| Li (2020) | Abdominal bloating | Not serious (0) | Not serious (0) | Not serious (0) | Serious (−1) | Serious (−1) | LOW ⊕⊕⊖⊖ |
| Sun (2020) | Abdominal pain | Not serious (0) | Serious (−1) | Serious (−1) | Not serious (0) | Not serious (0) | LOW ⊕⊕⊖⊖ |
|  | Abdominal bloating | Not serious (0) | Serious (−1) | Not serious (0) | Serious (−1) | Unclear (0) | LOW ⊕⊕⊖⊖ |
|  | Quality of life | Not serious (0) | Uncertain | Not serious (0) | Very serious (−2) | Unclear (0) | LOW ⊕⊕⊖⊖ |
| Dale (2019) | Abdominal pain | Not serious (0) | Serious (−1) | Not serious (0) | Serious (−1) | Unclear (0) | LOW ⊕⊕⊖⊖ |
|  | Abdominal bloating | Not serious (0) | Serious (−1) | Not serious (0) | Serious (−1) | Unclear (0) | LOW ⊕⊕⊖⊖ |
|  | Quality of life | Not serious (0) | Serious (−1) | Not serious (0) | Serious (−1) | Unclear (0) | LOW ⊕⊕⊖⊖ |
| Connell (2018) | Abdominal pain | Serious (−1) | Not serious (0) | Not serious (0) | Serious (−1) | Not serious (0) | LOW ⊕⊕⊖⊖ |
|  | Abdominal bloating | Serious (−1) | Not serious (0) | Not serious (0) | Serious (−1) | Not serious (0) | LOW ⊕⊕⊖⊖ |
|  | Quality of life | Serious (−1) | Not serious (0) | Not serious (0) | Serious (−1) | Not serious (0) | LOW ⊕⊕⊖⊖ |
| Ford (2018) | General symptoms | Not serious (0) | Serious (−1) | Not serious (0) | Not serious (0) | Serious (−1) | LOW ⊕⊕⊖⊖ |
|  | Abdominal bloating | Serious (−1) | Not serious (0) | Not serious (0) | Serious (−1) | Unclear (0) | LOW ⊕⊕⊖⊖ |
| Yuan (2017) | Abdominal pain | Not serious (0) | Serious (−1) | Not serious (0) | Serious (−1) | Unclear (0) | LOW ⊕⊕⊖⊖ |
| Didari (2015) | General symptoms | Not serious (0) | Serious (−1) | Not serious (0) | Serious (−1) | Not serious (0) | LOW ⊕⊕⊖⊖ |
|  | Abdominal bloating | Not serious (0) | Not serious (0) | Serious (−1) | Very serious (−2) | Not serious (0) | VERY LOW ⊕⊖⊖⊖ |
|  | Quality of life | Not serious (0) | Serious (−1) | Serious (−1) | Not serious (0) | Not serious (0) | LOW ⊕⊕⊖⊖ |
| Moayyedi (2010) | General symptoms (dichotomous) | Not serious (0) | Serious (−1) | Not serious (0) | Not serious (0) | Serious (−1) | LOW ⊕⊕⊖⊖ |
|  | Abdominal bloating | Not serious (0) | Serious (−1) | Not serious (0) | Serious (−1) | Not serious (0) | LOW ⊕⊕⊖⊖ |
| Brenner (2009) | Quality of life | Serious (−1) | Not serious (0) | Not serious (0) | Serious (−1) | Not serious (0) | LOW ⊕⊕⊖⊖ |
| Hoveyda (2009) | Abdominal bloating | Serious (−1) | Not serious (0) | Not serious (0) | Serious (−1) | Not serious (0) | LOW ⊕⊕⊖⊖ |
|  | Quality of life | Serious (−1) | Not serious (0) | Not serious (0) | Serious (−1) | Not serious (0) | LOW ⊕⊕⊖⊖ |

**Critical items for confidence in evidence (GRADE)**

GRADE rates the certainty/confidence of evidence per outcome on four levels (High, Moderate, Low, Very low) combining 5 domains:

**Downgrading domains:**

• **Risk of bias (RoB)**: serious limitations of studies contributing to the outcome (selection, performance, detection, attrition, selective reporting bias).

• **Inconsistency**: unexplained substantive heterogeneity (disparate effects, high I², prediction interval breadth crossing null, opposite directions).

• **Indirectness**: relevant differences in PICO (population, intervention/comparator, outcome, or context) or use of surrogates.

• **Imprecision**: insufficient effective sample size or wide 95%CI including both clinically relevant benefits and harms.

• **Publication bias/small studies**: funnel plot asymmetry, exaggerated results in small studies, missing gray literature.

**Global Rating Criteria:**

• **High**: very high confidence; new evidence very unlikely to change the estimate. (RCTs without important limitations; 0 downgraded domains).

• **Moderate**: moderate confidence; new evidence could change the estimate. (1-level downgrade in ≥1 domain).

• **Low**: limited confidence; new evidence likely to change the estimate. (2-level downgrade in 1 domain or 1-level in ≥2 domains).

• **Very low**: great uncertainty; the estimate is very uncertain. (≥3 cumulative downgrade levels).
