# Supplementary material for: Probiotics in Irritable Bowel Syndrome: An Umbrella Review of 27 Systematic Reviews on Methodological Quality and Certainty of Evidence
Source: J Clin Med. 2026 Feb 25;15(5):1727. doi: 10.3390/jcm15051727 (PMC12985868; doi:10.3390/jcm15051727)
Supplement: Supplementary file 1 [file jcm-15-01727-s001.zip › Supplementary Material/Table S6.docx]

**Supplementary Material Table 6.** Complete evaluation of clinical efficacy and methodological quality of the 27 included systematic reviews

|  | **General symptoms** | | **Abdominal pain** | | **Abdominal bloating** | | **Quality of life** | |
| --- | --- | --- | --- | --- | --- | --- | --- | --- |
|  | Result | GRADE classification | Result | GRADE classification | Result | GRADE classification | Result | GRADE classification |
| Yu Q-X (2025) | OR 5.09 (95%CI 4.19–6.20) | VERY LOW ⊕⊖⊖⊖ | _ | _ | _ | _ | _ | _ |
| Almabruk (2024) | Global relief reported in most studies | VERY LOW ⊕⊖⊖⊖ | SMD: -1.66 (95%CI −2.39 to −0.93). | VERY LOW ⊕⊖⊖⊖ | SMD: -2.13 (95%CI: -3.96 to -0.30) | VERY LOW ⊕⊖⊖⊖ | Improvement of +8.77 points (95%CI: +0.91 to +16.64) | VERY LOW ⊕⊖⊖⊖ |
| Wu (2024) | OR 0.53 (95%CI 0.48–0.59)<br>SMD −0.48 (95%CI −0.62 to −0.35) | MODERATE (⊕⊕⊕⊖) | SMD −0.46 (−0.79 to −0.14) | VERY LOW ⊕⊖⊖⊖ | SMD: not significant | LOW ⊕⊕⊖⊖ | SMD −0.35 (−0.54 to −0.15) | MODERATE ⊕⊕⊕⊖ |
| Umeano (2024) | 7/8 RCTs conclude that probiotics may improve | LOW ⊕⊕⊖⊖ | Several RCTs showed benefits | VERY LOW ⊕⊖⊖⊖ | Some studies found benefit in bloating and flatulence. | VERY LOW ⊕⊖⊖⊖ | Several RCTs showed improvements (IBS-QoL/SF-36), although without aggregate magnitude. | LOW ⊕⊕⊖⊖ |
| Yang (2024) | RR 1.40 (95%CI: 1.18–1.66)<br>Improvement SMD = 0.4 (95%CI 0.17–0.26). | MODERATE ⊕⊕⊕⊖ | Improvement SMD = 0.39 (95%CI 0.12–0.65). | LOW ⊕⊕⊖⊖ | SMD: not significant | LOW ⊕⊕⊖⊖ | Improvement: SMD 0.29 (95%CI 0.15–0.42) | LOW ⊕⊕⊖⊖ |
| Chen (2023) | SMD −0.55 (95%CI −0.76 to −0.34) | LOW ⊕⊕⊖⊖ | SMD −0.89 (95%CI −1.29; −0.50) | LOW ⊕⊕⊖⊖ | SMD −0.77 (95%CI −1.20 to −0.35) | LOW ⊕⊕⊖⊖ | SMD 0.99 (95%CI: 0.45 to 1.54). | VERY LOW ⊕⊖⊖⊖ |
| Goodoory (2023) | RR 0.78 (95%CI 0.71–0.87) | VERY LOW ⊕⊖⊖⊖ | RR 0.72 (95%CI 0.64–0.82) | VERY LOW ⊕⊖⊖⊖ | RR 0.75 (95%CI 0.64–0.88), | VERY LOW ⊕⊖⊖⊖ | _ | _ |
| Qing (2023) | _ | _ | SMD = −0.205 (95%CI: −0.347 to −0.063) | MODERATE ⊕⊕⊕⊖ | SMD = not significant | MODERATE ⊕⊕⊕⊖ | 2/3 RCTs showed modest improvements | LOW ⊕⊕⊖⊖ |
| Xie (2023) | MD −77.70 (95%CI: −101.72 to −53.68) | MODERATE ⊕⊕⊕⊖ | SMD −41.80 (95%CI: −61.59 to −22.00) | MODERATE ⊕⊕⊕⊖ | SMD −34.00 (95%CI: −56.94 to −11.06) | MODERATE ⊕⊕⊕⊖ | MD +15.35 (95%CI: +4.45 to +26.26) | MODERATE ⊕⊕⊕⊖ |
| Konstantis (2023) | WMD: not significant | VERY LOW ⊕⊖⊖⊖ | SMD: −0.94 (95%CI −1.53 to −0.35) | LOW ⊕⊕⊖⊖ | SMD: −0.28 (95%CI −0.47 to −0.09) | MODERATE ⊕⊕⊕⊖ | No significant differences | VERY LOW ⊕⊖⊖⊖ |
| Wang (2022) | SMD −0.55 (95%CI −0.83 to −0.27) | VERY LOW ⊕⊖⊖⊖ | SMD −0.43 (95%CI −0.57 to −0.29) | VERY LOW ⊕⊖⊖⊖ | SMD −0.45 (95%CI −0.81 to −0.09) | VERY LOW ⊕⊖⊖⊖ | No significant differences | VERY LOW ⊕⊖⊖⊖ |
| Van der Geest (2022) | RR 0.68 (95%CI 0.51–0.92) | LOW ⊕⊕⊖⊖ | SMD −0.35 (95%CI −0.56 to −0.14) | MODERATE ⊕⊕⊕⊖ | No significant differences | LOW ⊕⊕⊖⊖ | _ | _ |
| Shang (2022) | _ | _ | No significant differences | LOW ⊕⊕⊖⊖ | No significant differences | LOW ⊕⊕⊖⊖ | No significant differences | LOW ⊕⊕⊖⊖ |
| Xie (2022) | RR = 1.50 (95%CI 1.10–2.05) | MODERATE ⊕⊕⊕⊖ | SMD = −1.51 (95%CI −2.18 to −0.85) | LOW ⊕⊕⊖⊖ | _ | _ | _ | _ |
| Wen (2020) | RR = not significant | LOW ⊕⊕⊖⊖ | _ | _ | SMD −0.77 (95%CI −1.46 to −0.07) | MODERATE ⊕⊕⊕⊖ | _ | _ |
| Li (2020) | RR 1.52 (95%CI 1.32–1.76)<br>SMD −0.18 (95%CI −0.30 to −0.06) | MODERATE ⊕⊕⊕⊖ | SMD −0.22 (95%CI −0.33 to −0.11) | MODERATE ⊕⊕⊕⊖ | SMD −0.13 (95%CI −0.24 to −0.03) | LOW ⊕⊕⊖⊖ | _ | _ |
| Niu (2020) | RR = 0.79 (95%CI 0.70–0.89) | MODERATE ⊕⊕⊕⊖ | SMD −0.25 (95%CI −0.36 to −0.14) | MODERATE ⊕⊕⊕⊖ | SMD −0.15 (95%CI −0.27 to −0.03) | MODERATE ⊕⊕⊕⊖ | _ | _ |
| Sun (2020) | RR 1.50 (95%CI 1.23–1.83) | MODERATE ⊕⊕⊕⊖ | SMD −0.31 (95%CI −0.45 to −0.17) | LOW ⊕⊕⊖⊖ | SMD −0.20 (95%CI −0.38 to −0.01) | LOW ⊕⊕⊖⊖ | SMD: not significant | LOW ⊕⊕⊖⊖ |
| Dale (2019) | 7/10 RCTs showed clinically relevant improvement | MODERATE ⊕⊕⊕⊖ | 5/7 RCTs showed significant benefit | LOW ⊕⊕⊖⊖ | 5/7 studies show consistent improvement | LOW ⊕⊕⊖⊖ | Improvement in 3 RCTs of 6 total RCTs | LOW ⊕⊕⊖⊖ |
| Liang (2019) | RR 1.27 (95%CI 1.13–1.44) | HIGH ⊕⊕⊕⊕ | _ | _ | _ | _ | _ | _ |
| Connell (2018) | RR: not significant. | MODERATE ⊕⊕⊕⊖ | SMD: not significant | LOW ⊕⊕⊖⊖ | SMD: not significant. | LOW ⊕⊕⊖⊖ | SMD: not significant. | LOW ⊕⊕⊖⊖ |
| Ford (2018) | RR 0.79 (95%CI 0.68–0.91) | LOW ⊕⊕⊖⊖ | SMD −0.31 (95%CI −0.44 to −0.17) | MODERATE ⊕⊕⊕⊖ | SMD −0.13; 95%CI −0.34 to −0.01 | LOW ⊕⊕⊖⊖ | _ | _ |
| Yuan (2017) | _ | _ | SMD: not significant. | LOW ⊕⊕⊖⊖ | SMD = 0.21 (95%CI 0.07 to 0.35) | MODERATE ⊕⊕⊕⊖ | _ | _ |
| Didari (2015) | RR = 2.14 (95%CI 1.08–4.26) | LOW ⊕⊕⊖⊖ | SMD: not significant | MODERATE ⊕⊕⊕⊖ | SMD: not significant | VERY LOW ⊕⊖⊖⊖ | 3/5 RCTs show improvement; others do not | LOW ⊕⊕⊖⊖ |
| Moayyedi (2010) | RR = 0.71; 95%CI 0.57–0.88<br>SMD −0.34 (95%CI −0.60 to −0.07) | LOW ⊕⊕⊖⊖ | SMD = −0.51 (95%CI −0.91 to −0.09) | MODERATE ⊕⊕⊕⊖ | SMD = −0.54 (95%CI −1.10 to 0.02) | LOW ⊕⊕⊖⊖ | _ | _ |
| Brenner (2009) | B. infantis 35624: 2/2 RCTs show benefit | MODERATE ⊕⊕⊕⊖ | B. infantis 35624: 2/2 RCTs show reduction | MODERATE ⊕⊕⊕⊖ | B. infantis 35624: 2/2 RCTs report decrease | MODERATE ⊕⊕⊕⊖ | Lactobacillus reuteri ATCC 55730: 1/1 shows no difference. | LOW ⊕⊕⊖⊖ |
| Hoveyda (2009) | OR 1.59 (95%CI 1.19–2.13) | MODERATE ⊕⊕⊕⊖ | OR 3.34 (95%CI 1.99–5.61) | MODERATE ⊕⊕⊕⊖ | OR 1.75 (95%CI 1.03–2.96) | LOW ⊕⊕⊖⊖ | No significant differences | LOW ⊕⊕⊖⊖ |
