# Supplementary material for: Probiotics in Irritable Bowel Syndrome: An Umbrella Review of 27 Systematic Reviews on Methodological Quality and Certainty of Evidence
Source: J Clin Med. 2026 Feb 25;15(5):1727. doi: 10.3390/jcm15051727 (PMC12985868; doi:10.3390/jcm15051727)
Supplement: Supplementary file 1 [file jcm-15-01727-s001.zip › Supplementary Material/Table S7.docx]

**Supplementary Material Table 7:** AMSTAR-2 methodological quality assessment

| **Author (year)** | **1** | **2** | **3** | **4** | **5** | **6** | **7** | **8** | **9** | **10** | **11** | **12** | **13** | **14** | **15** | **16** | **AMSTAR-2 result** |
| --- | --- | --- | --- | --- | --- | --- | --- | --- | --- | --- | --- | --- | --- | --- | --- | --- | --- |
| Yu Q-X (2025) | Yes | Partial | Partial | Partial | Yes | Yes | No | Yes | Yes | No | Yes | Partial | Yes | Yes | Yes | Yes | Low |
| Almabruk (2024) | Partial | No | Partial | No | Yes | No | No | Yes | Yes | No | No | No | Partial | Partial | Yes | Yes | Critically low |
| Wu (2024) | Yes | Yes | Partial | Yes | Yes | Yes | No | Yes | Yes | No | Yes | No | Yes | Yes | Yes | Yes | Low |
| Umeano (2024) | Yes | No | Partial | Partial | No | No | No | Yes | No | No | Not applicable | Not applicable | No | Not applicable | Not applicable | Yes | Critically low |
| Yang (2024) | Yes | Yes | Partial | Partial | No | No | No | Yes | Yes | No | Yes | Partial | Partial | Yes | Yes | Yes | Low |
| Chen (2023) | Yes | Yes | Partial | Yes | Yes | Yes | No | Yes | Yes | No | Yes | Partial | Yes | Yes | Yes | Yes | Low |
| Goodoory (2023) | Yes | No | Partial | Yes | Yes | Yes | No | Yes | Yes | No | Yes | Yes | Yes | Yes | Yes | Yes | Critically low |
| Qing (2023) | Yes | No | Partial | Partial | Yes | Yes | No | Yes | Yes | No | Partial | Partial | Partial | Partial | No | Yes | Critically low |
| Xie (2023) | Yes | Yes | Partial | Yes | Yes | Yes | No | Yes | Yes | Partial | Yes | Partial | Partial | Yes | Partial | Yes | Low |
| Konstantis (2023) | Yes | Yes | Partial | Yes | Yes | Yes | No | Yes | Yes | No | Yes | Yes | Yes | Partial | Not applicable | Yes | Low |
| Wang (2022) | Yes | No | Partial | Yes | Yes | Yes | No | Yes | Yes | Partial | Yes | Partial | Yes | Yes | Partial | Yes | Critically low |
| Van der Geest (2022) | Yes | No | Partial | No | Partial | No | No | Partial | No | No | Yes | No | Partial | Yes | Yes | Yes | Critically low |
| Shang (2022) | Yes | No | Partial | Partial | Yes | Partial | No | Yes | Yes | No | Yes | Partial | Yes | Yes | Partial | Yes | Critically low |
| Xie (2022) | Yes | No | Partial | Partial | Yes | Partial | No | Yes | Yes | No | Yes | Partial | Yes | Yes | No | Yes | Critically low |
| Wen (2020) | Yes | Partial | Partial | Partial | Yes | Yes | No | Yes | Yes | No | Yes | Partial | Yes | Yes | Partial | Yes | Low |
| Li (2020) | Yes | No | Partial | Partial | Yes | Yes | No | Yes | Yes | No | Yes | Yes | Yes | Yes | Yes | Yes | Critically low |
| Niu (2020) | Yes | Partially | Partial | Partial | Yes | Yes | No | Yes | Yes | No | Yes | Yes | Yes | Yes | Yes | Yes | Low |
| Sun (2020) | Yes | Yes | Yes | Partial | Yes | Yes | No | Yes | Yes | No | Yes | Yes | Yes | Yes | Yes | Yes | Low |
| Dale (2019) | Yes | No | Partial | No | No | No | No | Yes | No | No | Not applicable | Not applicable | No | Partial | Not applicable | Yes | Critically low |
| Liang (2019) | Yes | No | Partial | Partial | No | Yes | No | Yes | Yes | No | Yes | Partial | Partial | Yes | Yes | Yes | Critically low |
| Connell (2018) | Yes | No | Partial | Yes | Yes | Yes | No | Yes | Yes | No | Yes | Partial | Yes | Partial | Partial | Yes | Critically low |
| Ford (2018) | Yes | No | Partial | Yes | Yes | Yes | No | Yes | Yes | No | Yes | Partial | Yes | Yes | Yes | Yes | Critically low |
| Yuan (2017) | Yes | No | Partial | Partial | Yes | No | No | Yes | Partial | No | Yes | No | No | Partial | Partial | Yes | Critically low |
| Didari (2015) | Yes | No | Partial | Partial | Partial | No | No | Yes | Partial | No | Yes | No | Partial | Partial | Partial | Yes | Critically low |
| Moayyedi (2010) | Yes | No | Yes | Yes | Yes | Yes | No | Yes | Partial | No | Yes | Yes | Yes | Yes | Yes | Yes | Critically low |
| Brenner (2009) | Yes | No | Partial | Partial | Yes | Yes | No | Yes | Partial | No | Partial | Partially | Yes | Partial | Not applicable | Yes | Critically low |
| Hoveyda (2009) | Yes | No | Partial | Partial | Yes | Yes | No | Yes | Partial | No | Partial | Yes | Yes | Yes | Partial | Yes | Critically low |

**Critical items for methodological validity**

The assessment identifies seven critical elements that determine the quality of a systematic review. Prospective protocol registration (item 2) ensures transparency and reduces selective reporting bias. A comprehensive literature search (item 4), reproducible and up-to-date, minimizes omissions. The list of excluded studies with justification (item 7) allows assessment of screening rigor and rules out selection bias. Risk of bias assessment in studies (item 9) is indispensable for interpreting evidence. Use of appropriate meta-analysis methods (item 11) supports the validity of quantitative conclusions. Integration of risk of bias in interpretation (item 13) conditions confidence in findings. Finally, assessment of publication bias (item 15) detects unreported evidence that could alter conclusions.

**Non-critical but relevant items**

Non-critical items complement methodological robustness: PICO formulation (item 1) defines the question framework; justification of eligible designs (item 3) clarifies criteria; duplicate selection (item 5) and duplicate extraction (item 6) reduce errors; detailed description of included studies (item 8) facilitates reproducibility; reporting of primary study funding (item 10) identifies possible conflicts; assessment of risk of bias impact on synthesis results (item 12) reinforces interpretation; exploration and explanation of heterogeneity (item 14) contextualizes variability; and declaration of review authors' conflicts of interest (item 16) ensures transparency.

**Global Rating Criteria:**

• **High**: No critical weaknesses or up to one non-critical weakness

• **Moderate**: More than one non-critical weakness; the review provides an accurate and comprehensive summary of results from available studies

• **Low**: One critical weakness with or without non-critical weaknesses

• **Critically Low**: More than one critical weakness with or without other weaknesses

**Scoring:** Yes = Fully meets; Partial = Partially meets; No = Does not meet; N/A = Not applicable (for narrative reviews without meta-analysis); Not reported = Information not available
