# Supplementary material for: Probiotics in Irritable Bowel Syndrome: An Umbrella Review of 27 Systematic Reviews on Methodological Quality and Certainty of Evidence
Source: J Clin Med. 2026 Feb 25;15(5):1727. doi: 10.3390/jcm15051727 (PMC12985868; doi:10.3390/jcm15051727)
Supplement: Supplementary file 1 [file jcm-15-01727-s001.zip › Supplementary Material/Table S8.docx]

**Supplementary Material Table 8.** ROBIS risk of bias assessment

| **Author (year)** | **Domain 1** | **Domain 2** | **Domain 3** | **Domain 4** | **ROBIS result** |
| --- | --- | --- | --- | --- | --- |
| Yu Q-X (2025) | High risk of concern | High risk of concern | High risk of concern | High risk of concern | High risk of bias |
| Almabruk (2024) | High risk of concern | High risk of concern | Unclear | High risk of concern | High risk of bias |
| Wu (2024) | Low risk of concern | Low risk of concern | High risk of concern | High risk of concern | High risk of bias |
| Umeano (2024) | Unclear | High risk of concern | High risk of concern | High risk of concern | High risk of bias |
| Yang (2024) | High risk of concern | High risk of concern | Unclear | Unclear | High risk of bias |
| Chen (2023) | Low risk of concern | Unclear | Low risk of concern | High risk of concern | High risk of bias |
| Goodoory (2023) | Low risk of concern | Unclear | Low risk of concern | Unclear | Unclear |
| Qing (2023) | Unclear | Low risk of concern | Low risk of concern | High risk of concern | High risk of bias |
| Xie (2023) | Low risk of concern | Low risk of concern | Low risk of concern | Unclear | Unclear |
| Konstantis (2023) | Low risk of concern | Low risk of concern | Low risk of concern | High risk of concern | High risk of bias |
| Wang (2022) | Low risk of concern | Unclear | Unclear | Unclear | Unclear |
| van der Geest (2022) | Unclear | High risk of concern | High risk of concern | High risk of concern | High risk of bias |
| Shang (2022) | High risk of concern | High risk of concern | Low risk of concern | High risk of concern | High risk of bias |
| Xie (2020) | Unclear | High risk of concern | Unclear | High risk of concern | High risk of bias |
| Wen (2020) | High risk of concern | Unclear | Low risk of concern | High risk of concern | High risk of bias |
| Li (2020) | High risk of concern | Low risk of concern | Low risk of concern | High risk of concern | High risk of bias |
| Niu (2020) | Low risk of concern | High risk of concern | Low risk of concern | Unclear | High risk of bias |
| Sun (2020) | Low risk of concern | Unclear | Low risk of concern | High risk of concern | High risk of bias |
| Dale (2019) | Unclear | High risk of concern | High risk of concern | High risk of concern | High risk of bias |
| Liang (2019) | High risk of concern | High risk of concern | Unclear | High risk of concern | High risk of bias |
| Connell (2018) | Low risk of concern | High risk of concern | Low risk of concern | Unclear | High risk of bias |
| Ford (2018) | Low risk of concern | Unclear | Low risk of concern | Unclear | Unclear |
| Yuan (2017) | High risk of concern | High risk of concern | High risk of concern | High risk of concern | High risk of bias |
| Didari (2015) | High risk of concern | High risk of concern | High risk of concern | High risk of concern | High risk of bias |
| Moayyedi (2010) | Low risk of concern | Low risk of concern | Low risk of concern | High risk of concern | High risk of bias |
| Brenner (2009) | High risk of concern | High risk of concern | Unclear | High risk of concern | High risk of bias |
| Hoveyda (2009) | High risk of concern | Low risk of concern | Low risk of concern | Unclear | High risk of bias |

**Critical items for methodological validity (ROBIS domains)**

ROBIS judges the risk of bias of a review across four domains, all critical for confidence in findings:

**Domain 1 (Eligibility criteria):** PICO and prespecified criteria applied consistently, without post-hoc restrictions.

**Domain 2 (Identification and selection of studies):** Comprehensive search (major databases, registries, gray literature), duplicate management, transparent screening (PRISMA flow), and justification of exclusions.

**Domain 3 (Data collection and study appraisal):** Duplicate extraction, handling of missing data, and appropriate risk of bias assessment of studies (valid tools and consistent use).

**Domain 4 (Synthesis and findings):** Adequate synthesis methods (or explicit narrative), heterogeneity assessment, predefined sensitivity/subgroup analyses, consideration of publication bias/small studies, and conclusions consistent with evidence and its limitations.

**Global rating criteria**

- **Low risk of bias:** Low concerns across all four domains and conclusions in line with quality/limitations of evidence.
- **High risk of bias:** High concern in ≥1 domain or conclusions that do not reflect detected limitations.
- **Unclear:** Insufficient information to judge one or more domains.
