# Supplementary figures and images for: Probiotics in Irritable Bowel Syndrome: An Umbrella Review of 27 Systematic Reviews on Methodological Quality and Certainty of Evidence
Source: J Clin Med. 2026 Feb 25;15(5):1727. doi: 10.3390/jcm15051727 (PMC12985868; doi:10.3390/jcm15051727)

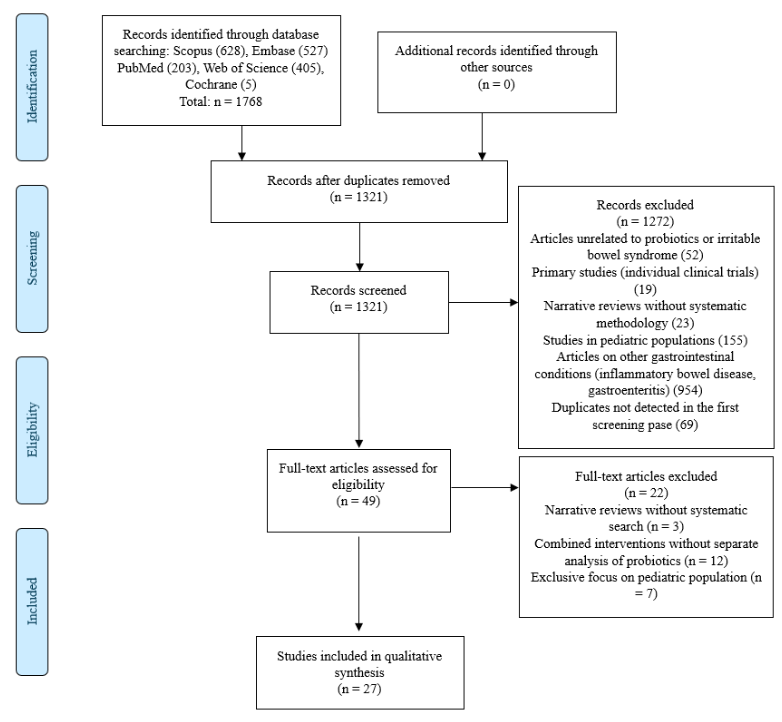

Supplement: Supplementary file 1 [file jcm-15-01727-s001.zip › Supplementary Material/FIGURE S1 .png]
